# Research Progress in Current and Emerging Issues of PFASs’ Global Impact: Long-Term Health Effects and Governance of Food Systems

**DOI:** 10.3390/foods14060958

**Published:** 2025-03-11

**Authors:** Jocelyn C. Lee, Slim Smaoui, John Duffill, Ben Marandi, Theodoros Varzakas

**Affiliations:** 1 Independent Researcher—Food Safety Consultant, San Francisco Bay Area, San Francisco, CA 94121, USA; jlee@gourmetrail.com; 2 Laboratory of Microbial and Enzymatic Biotechnologies and Biomolecules, Center of Biotechnology of Sfax (CBS), University of Sfax, Road of Sidi Mansour Km 6, P.O. Box 1177, Sfax 3018, Tunisia; slim.smaoui@cbs.rnrt.tn; 3 John Crop Development Vietnam Co., Ltd., Landmark 81, 720A Dien Bien Phu St., Binh Thanh Dist., Quận Bình Thạnh, Ho Chi Minh City 718900, Vietnam; john@jcdvn.com; 4 Food Scientist Researcher, Food Policy and Legal Advisor, 26 Lauren Beth Dr., Richmond Hill, ON L4E 4K3, Canada; bmarandi@outlook.com; 5 Department of Food Science and Technology, University of the Peloponnese, 24100 Kalamata, Greece

**Keywords:** PFAS, health risks, food safety, security, sustainability, systems failure

## Abstract

Per- and polyfluoroalkyl substances (PFASs) are found everywhere, including food, cosmetics, and pharmaceuticals. This review introduces PFASs comprehensively, discussing their nature and identifying their interconnection with microplastics and their impacts on public health and the environment. The human cost of decades of delay, cover-ups, and mismanagement of PFASs and plastic waste is outlined and briefly explained. Following that, PFASs and long-term health effects are critically assessed. Risk assessment is then critically reviewed, mentioning different tools and models. Scientific research and health impacts in the United States of America are critically analyzed, taking into consideration the Center for Disease Control (CDC)’s PFAS Medical Studies and Guidelines. PFAS impact and activities studies around the world have focused on PFAS levels in food products and dietary intake in different countries such as China, European countries, USA and Australia. Moreover, PFASs in drinking water and food are outlined with regard to risks, mitigation, and regulatory needs, taking into account chemical contaminants in food and their impact on health and safety. Finally, PFAS impact and activities briefings specific to regions around the world are discussed, referring to Australia, Vietnam, Canada, Europe, the United States of America (USA), South America, and Africa. The PFAS crisis is a multifaceted issue, exacerbated by mismanagement, and it is discussed in the context of applying the following problem-solving analytical tools: the Domino Effect Model of accident causation, the Swiss Cheese Theory Model, and the Ishikawa Fish Bone Root Cause Analysis. Last but not least, PFASs’ impacts on the Sustainable Development Goals (SDGs) of 2030 are rigorously discussed.

## 1. Introduction

### 1.1. PFASs’ Origins and Complacency

Perfluorooctanoic acid (PFOA) and PFASs are a group of synthetic chemicals that have been used since the 1940s. Due to their unique ability to repel oil and water, PFASs have been extensively utilized in a wide range of consumer products, including non-stick cookware, water-repellent clothing, stain-resistant fabrics, firefighting foams, grease-resistant food packaging, cosmetics, pharmaceutical containers, pesticides, and more [1].

Initially developed by chemical manufacturers like 3M and DuPont in the mid-20th century, PFASs became widely adopted in industrial and consumer products for their durability and resistance to chemical breakdown. Unfortunately, these same attributes have led to their persistence in the environment and human body, where they accumulate over time [1].

**Figure 1:**
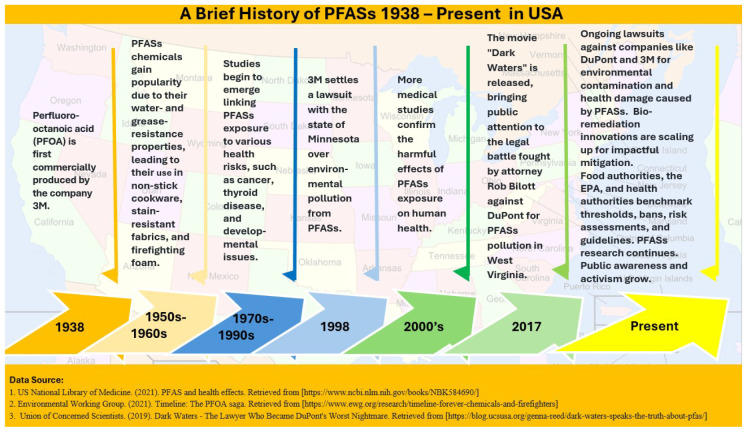
Timeline of PFASs development and impact (1938–present). Brief history from 1938 to present in USA [2,3,4].

The self-inflicted insidious invasive ubiquity of the PFAS crisis (1940s to present) indicates the development of PFASs firstly in the late 1930s and their wide usage in the 1940s for their unique properties, such as resistance to heat, water, and oil (Figure 1). These “forever chemicals” have since infiltrated various industries, including textiles, food packaging, and firefighting foams. Their persistence in the environment and the human body has led to widespread contamination, making them nearly ubiquitous in modern life [1].

Chemical giants like 3M, DuPont, and Chemours have played significant roles in the proliferation of PFASs. These companies have leveraged their economic power and political influence on shaping regulations and public perception, often downplaying the risks associated with PFASs. Their lobbying efforts have delayed stricter regulations and allowed continued production and use of these harmful substances [5].

The post-World War II era, often referred to as the “Golden Age of Capitalism”, saw rapid industrial growth and technological advancements. This period was marked by increased consumerism and the mass production of goods, including those containing PFASs. The economic boom led to the widespread use of synthetic chemicals without adequately considering their long-term environmental and health impacts [6].

For decades, the potential risks of PFASs were not adequately assessed. Regulatory frameworks lagged behind the rapid development and deployment of these chemicals. The lack of comprehensive risk assessments meant that PFASs were used extensively before their harmful effects were fully understood [7,8].

Major chemical companies, for decades, were aware of the potential health risks associated with PFAS exposure but often prioritized profits over safety [9]. Internal documents from companies like DuPont have revealed that, while they knew about the harmful effects of PFASs, they continued production, keeping the information hidden from the public. For example, there have been numerous instances where chemical companies suppressed scientific data on the health risks of PFASs. Internal documents from companies like 3M and DuPont revealed that they were aware of the dangers posed by PFASs as early as the 1960s but chose to conceal this information [5,10]. This cover-up has significantly delayed regulatory actions and public awareness [9].

Numerous lawsuits against chemical companies for PFAS contamination exist. A landmark case was settled in 2017 when DuPont and its spin-off Chemours agreed to pay USD 671 million to resolve 3550 personal injury claims linked to PFOA releases in Parkersburg, West Virginia. Similar litigations continue to emerge as states and municipalities seek damage reparations and funding for water treatment solutions [11].

### 1.2. The Nexus Between PFASs and Microplastics: An Emerging Threat to Public Health and the Environment

As we continue to assess the environmental toll of PFASs, another critical issue has emerged: their interactions with microplastics, further complicating their environmental impact [12].

PFASs and microplastics represent two significant environmental contaminants. While they have been studied individually, understanding their combined impact—the PFASs and microplastics nexus—is increasingly critical for public health, consumer awareness, and environmental sustainability [13,14].

PFASs are a group of synthetic chemicals extensively used in industrial processes and consumer products, including non-stick cookware, stain-resistant fabrics, and firefighting foams. They are highly resistant to degradation, earning them the moniker “forever chemicals”.

Microplastics are plastic fragments smaller than 5 mm, originating primarily from the degradation of larger plastic materials and the microbeads commonly found in personal care items. These particles have become widespread, contaminating oceans, freshwater systems, and soil, with evidence of their presence in human tissues and organs [15]. Microplastics can carry harmful chemicals, including PFASs, absorbing them from the surrounding environment [16].

Studies have revealed a critical interaction between microplastics and PFASs, where the former can act as carriers, helping PFASs move through and persist within different ecosystems [17]. The hydrophobic nature of microplastics enables them to absorb PFASs from contaminated water, leading to concentrated microplastics that act as new sources of PFAS pollution as they move through food webs [18]. This sorption process can lead to bioaccumulation and biomagnification, where organisms at higher trophic levels, including humans, are exposed to increased levels of these harmful substances [19].

The interaction between PFASs and microplastics poses severe public health concerns. Both contaminants are associated with a range of health issues. The presence of PFASs in microplastics can exacerbate their toxicity, leading to greater health risks upon ingestion or inhalation. This is particularly problematic in marine environments, where seafood consumption is a primary route of exposure to both microplastics and PFASs for humans [20].

The dual presence of PFASs and microplastics also brings heightened environmental challenges. PFAS-bound microplastics can disperse widely, affecting remote ecosystems and causing long-term environmental damage. They threaten wildlife, particularly aquatic organisms, by disrupting reproductive and hormonal systems and leading to physical harm from ingestion [21].

Raising consumer awareness about the sources and dangers of PFASs and microplastics is crucial. Individuals can reduce their exposure by avoiding products that contain these substances, such as certain cosmetics, non-stick cookware, and single-use plastics. Policy interventions, such as banning PFASs in consumer products and implementing stricter regulations on plastic use and disposal, are essential to mitigate this threat [22].

### 1.3. The Human Cost of Decades of Delay, Cover-Ups, and Mismanagement of PFASs and Plastic Waste

The delayed response and mismanagement of PFASs and plastic waste have had severe human costs. Communities near manufacturing sites have experienced higher rates of cancer, thyroid disease, and other health issues linked to PFAS exposure. The economic burden of healthcare costs and environmental cleanup is staggering, with estimates running into trillions of dollars [23,24,25].

The nexus between PFASs and microplastics represents a multifaceted environmental and public health challenge. As research continues to elucidate the complex interactions and impacts of these contaminants, informed policies and consumer practices are necessary to address the pervasive threat they pose to health and the environment.

## 2. PFASs and Long-Term Health Effects

While the environmental persistence of PFAS compounds raises substantial ecological concerns, their presence in the human body poses significant health risks, which have been increasingly documented in global health research.

PFASs possess a fluorinated carbon chain of different lengths, in partial or full form, terminated by a carboxylate or sulfonate, i.e., a functional head group [26,27,28]. Six or more fluorinated carbon backbones belong to long-chain PFASs, whereas less than six fluorinated carbons indicate the short-chain ones.

PFASs could induce reproductive toxicity, hepatotoxicity, and metabolic disorders [27,28,29,30,31,32,33], affecting the main organs such as the blood, liver, and kidneys.

Exposure to long-chain PFASs has been associated with risks of cardiovascular disease, immune system disorder, and cholesterol metabolisms, as indicated by epidemiological studies [34,35].

Hexafluoropropylene oxide-dimer acid (HFPO-DA), also known as GenX, constitutes a common short-chain PFAS alternative, replacing PFOA, the linear long-chain perfluorooctanoic acid representing most of the common PFASs, industrially, offering wide applications in manufacturing. These shorter-chain PFASs can accumulate in the human body and can be found everywhere [36,37,38,39].

Concentrations of serum PFOA and perfluorooctane sulfonic acid (PFOS) up to 32 μg/mL and 118 μg/mL, respectively, have been shown by occupationally exposed workers [30,40,41]. The average half-life of serum PFASs, such as PFOA and PFOS, has been evaluated to be in the range of 1–5 years. The reason for this long bio-accumulation and bio-persistence of long-chain PFASs in the circulation system is the most abundant protein in the blood, albumin, and its binding [40,41,42,43,44,45,46,47,48,49,50].

Peng et al. [51] reported that the branched short-chain GenX could bind to bovine serum albumin (BSA) with a lower affinity compared to that of linear long-chain PFOA.

The implications of PFASs affect children. The exposure of children to PFOA is associated with a higher risk of developing asthma. The same is true for exposure to PFOS, associated with impaired lung function as reported by Rafiee et al. [52].

A higher probability of exposure to PFASs appears in young children compared to adults due to their smaller size, higher respiratory rates, and behaviors related to hand-to-mouth and crawling which require interactions with contaminated surfaces, such as floors [53]. Inhalation; ingestion of dust, soil, food, water, and breast milk; and dermal exposure to contaminated air or materials consist of some of the major routes of children’s exposure to PFASs [54]. Higher concentrations of PFASs in the serum of children compared to adults have also been reported [55,56].

In the same context, Rocabois et al. [57] identified the dose–response relationships for 50 substance–outcome pairs, corresponding to 20 chemicals and 17 health outcomes. PFASs could contribute to cardiovascular disease [58].

Human induced pluripotent stem cell (iPSC)-derived cardiomyocytes are employed widely for cardiotoxicity testing. A total of 56 PFASs from different subclasses were tested in concentration–response tests using human iPSC-derived cardiomyocytes from 16 donors without known heart disease. Ford et al. [59] reported that of the tested PFASs, 46 showed concentration–response effects in at least one phenotype and donor.

PFASs are one of the most significant and highly persistent endocrine-disrupting chemicals (EDCs), with extremely high thermal and chemical stability [60,61]. Exposure to EDCs may modify the homeostasis of the endocrine system [62,63], which is affected by hormonal, genetic, immunological, lifestyle, and environmental factors [64]. Exposure to certain PFASs may increase the odds of endometriosis, as reported by de Haro-Romero et al. [65].

Multiple possible routes of exposure to PFASs exist, and these consist of the drinking water route and the diet [66,67,68,69]. PFASs have been detected in human placenta, breast milk, follicular fluid, and meconium samples [70,71,72,73,74]. Highly detectable serum or plasma concentrations of PFASs, such as PFOS, PFOA, perfluorononanoic acid (PFNA), and perfluorohexane sulfonate (PFHxS), have been found in pregnant women and children [74,75,76,77,78,79].

In addition, a positive association between certain PFASs and periodontitis, which might be partially mediated by sex hormones (testosterone and the ratio of testosterone to estradiol), has been shown by Wu et al. [80].

### 2.1. PFASs Around the Globe and Risk Assessment

Different studies have been carried out in the United States showing the correlation of serum PFAS concentration with hyperlipidemia. In the study by Zhou et al. [81], data from the 2013–2016 National Health and Nutrition Examination Survey were analyzed for a total of 2665 adults, taking into account participants’ serum PFASs (perfluorooctanoic acid [PFOA], PFNA, perfluorodecanoic acid (PFDA), (PFHxS), and PFOS).

In India, Koulini and Nambi [82] revealed PFAS levels up to 136.27 ng/L in both surface and groundwater samples from Chennai. The significant sources of contamination with PFASs turned out to be industrial emissions, untreated domestic wastewater discharge, and open dump sites. Hence, concerns were raised about potential risks to ecosystems and human well-being.

In Israel, Belmaker et al. [83] reported that there is likely no safe level of exposure to EDCs, with increasing evidence of trans-generational and epigenetic effects. They mentioned several existing Israeli laws to reduce plastic use and waste and suggested reinforcing the taxes on single-use plastic (SUP).

In Italy, Biggeri et al. [84] found an association of PFAS exposure with mortality from cardiovascular disease. They also showed evidence regarding kidney cancer and testicular cancer found to be consistent with previously reported data. The data came from the Italian National Institute of Health, which pre-processed and made available anonymous data from the Italian National Institute of Statistics death certificate archives for residents of the provinces of Vicenza, Padua, and Verona who died between 1980 and 2018, observing 51,621 deaths vs. 47,731 expected.

According to the European Food Safety Authority (EFSA), one of the most concerning health effects of PFAS exposure is the reduced antibody response to vaccines in young children. To address this, the EFSA established a tolerable weekly intake (TWI) level of 4.4 ng per kilogram of body weight for the combined total of four major PFAS compounds—PFOA, PFOS, PFNA, and PFHxS—commonly found in human serum [85].

PFAS risk assessment also includes modern computational tools, such as the Agent-Based Model (ABM) and Physiologically Based Kinetic (PBK) models serving as vital tools in chemical risk assessments, showing the interaction with the human immune system and enabling the simulation of host immune systems’ reactions to diverse stimuli and responses to specific adverse health contexts [86].

By taking into account (i) demographic factors, (ii) the local environment, (iii) individuals’ social behavior, (iv) and interaction with other individuals based on time–activity pattern data, the ABM could be a proper tool to understand the contact crossways time and space exposure [87]. The ABM simulates the performances and exchanges of independent agents within a system, permitting us to standardize these patterns at the scale of individuals and discover how behavior, demography, and social factors have an impact on damaging chemicals such as PFASs [88]. The ABM provides numerous advantages. It can be hypothetical, joining intervals and spatial explanations, which permits a more precise picture of the biological features and performances of the complicated trials. Additionally, the nonlinear comportments and the facility to assimilate added complexity and biological perceptions are simply enabled within the model. Consequently, the precision of the description is more often controlled by biological thought than by the modeling method.

PBK models, a series of mathematical equations, describe the absorption, distribution, metabolism, and excretion (ADME) features of a compound within an organism, and can envisage the physiologically pertinent levels of a compound in plasma or, when pertinent, in human tissue, all for a specified dose, time point, and path of administration. In the field of PFAS toxicity, PBK models have been employed to explore in vitro to in vivo estimation, interpreting in vitro data on toxicity or genotoxicity [89]. In addition, the in vivo acute liver toxicity or in vivo genotoxicity of PFASs could be assessed. To define the biokinetics of PFHxS and PFNA, PBK models of PFOS and PFOA were projected. The PBK models were adjusted concerning the PBK transporter maximum capacity for renal tubular reabsorption to reach the reported human elimination of half-lives of PFNA and PFHxS. The mean elimination half-lives of 8.2 and 3.2 years were applied for and PFHxS PFNA [90].

### 2.2. The Scientific Research and Health Impacts in the United States of America

Continued deep studies in the United States have confirmed the links between PFAS exposure and various health issues, including liver damage, thyroid disease, decreased fertility, high cholesterol, obesity, hormone suppression, and cancer. Academic research such as the C8 Science Panel, which studied the effects of PFOA (a type of PFAS) on communities near DuPont’s Washington Works plant, confirmed the associations between PFAS exposure and several health conditions.

The National Health and Nutrition Examination Survey (NHANES) has measured PFAS levels in blood in the USA population since 1999. The NHANES is a program of studies designed by the CDC to evaluate the health and nutrition of adults and children in the United States. NHANES data are publicly released in 2-year cycles [91].

**Figure 2:**
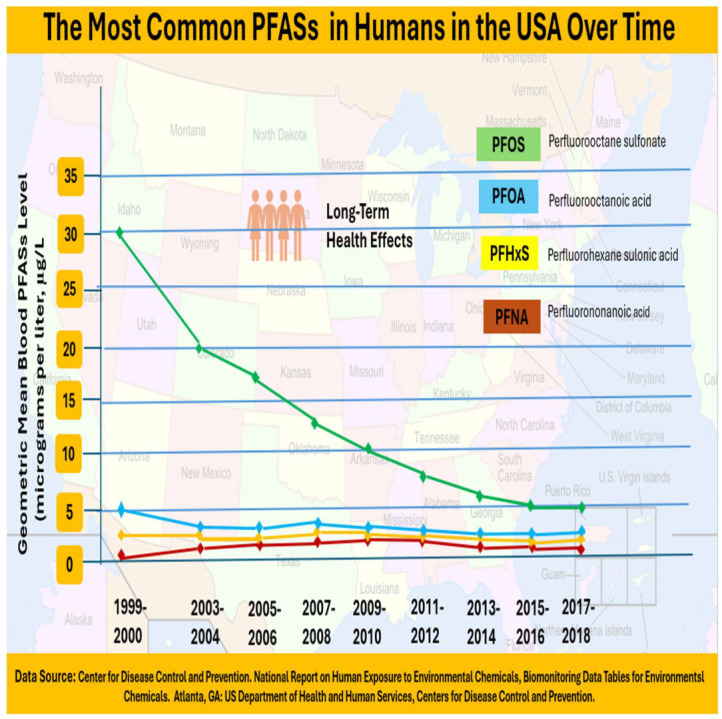
Blood levels of common PFASs in people, USA.

As depicted in Figure 2, since 2002, the manufacturing and consumption of PFOS and PFOA in the USA have decreased. Reduced PFAS use has led to lower blood PFAS levels. Blood PFOS levels fell by more than 85% between 1999–2000 and 2017–2018. Between 1999–2000 and 2017–2018, blood PFOA levels fell by more than 70%. As PFOS and PFOA are phased out and replaced, individuals may be exposed to additional PFASs [92]. The CDC has conducted and supported various studies related to PFASs, focusing on health impacts. The key findings and recommendations are included, as presented in Table 1.

## 3. PFAS Impact, Activities, and Dietary Intake Studies Around the World

In a general sense, most of the recent investigations have been performed in China, and European countries, and only some have been conducted in the USA and Australia.

### 3.1. China

Aquatic organisms, living in contaminated (artificial or natural) environments, are prone to accrue the pollutant-exceeding water environment in which they live. In this way, PFASs may be transmitted from contaminated water, food, or suspended sediment, with emphasis on the aquatic organism [98]. Wang et al. [99] examined the levels and human exposure to ten linear PFASs, eight branched PFASs for PFOA, comprising NaDONA, HFPO-DA, and PFOS, and four alternatives characterized in animal-origin and vegetable dietary food samples consumed by Beijing populaces. In this study, PFAS occurrence and concentrations in fish (freshwater and marine) samples were superior to other food trials. In these samples, linear PFASs were commonly found at 444 pg/g ww and 451 pg/g ww in marine and freshwater fish, respectively. The residents occupying Beijing, where fish consumption was the dominant source of ∑PFOS and 6:2 chlorinated polyfluorinated ether sulfonate (6:2 Cl-PFESA), were not meaningfully exposed to PFASs.

Via high-performance liquid chromatography–tandem mass spectrometry (HPLC-MS/MS), Bao et al. [100] investigated the extent of PFAS contamination in numerous home gardens around the Fuxin fluorochemical industrial park (FIP), comprising groundwater from the public water systems and garden soils. In the groundwater beneath the Fuxin FIP, the maximum levels of Perfluorobutane sulfonate (PFBS) and PFOA were 21 and 2.5 µg/L, respectively. Furthermore, 67–87% of the ΣPFASs, comprising alternative perfluorobutanoic acid (PFBA) and PFBS, were found to be the main pollutants in vegetables and eggs from the residential gardens around the FIP. In this regard, it was also established that PFBA could penetrate garden-produced fruits and vegetables through the application of local PFAS-contaminated drinking water for irrigation [101]. These outcomes were confirmed by the high link between PFBA/PFOA/PFBS in local groundwater and those detected in home-produced vegetables.

Later, Bao et al. [102] investigated PFAS occurrence in groundwater and surface water at the Fuxin FIP, Liaoning Province, China, and reported that the prevalence of PFBS and PFOA in the groundwater samples, with the relative abundance of these compounds, was 24–25 times greater than that stated in 2009 [103]. PFBA, PFOA, and PFBS were the main PFASs in greenhouse soil samples (≈6.1, 6.8, and 46 ng/g), tomato (≈87, 1.7, and 13 ng/g), and cucumber (≈63, 2.6, and 15 ng/g), showing a connection with PFAS levels in groundwater samples, signifying that PFAS contaminations could be present in the soil and vegetables in the greenhouse through long-term groundwater irrigation [102]. In addition, these authors detected high bioaccumulation efficiencies (BAFs) for short-chain PFASs in vegetables. As an illustration, the BAFs of shorter-chain PFASs in greenhouse cucumbers and tomatoes were beyond those of longer-chain PFASs. To this point, via daily consumption, contact with PFBA, PFOA, and PFBS in cucumbers and tomatoes and from local greenhouses might not pose a health risk for the residents living near the FIP. Another study was conducted in Hangzhou City, Zhejiang Province, China, close to a landfill. The ΣPFA levels in the groundwater samples ranged between 17.3 and 163 ng/L, and the greatest plentiful element was PFBA, followed by PFOA, Perfluorinated acid (PFPeA), Perfluorohexanoic acid (PFHxA), and Perfluoroheptanoic acid (PFHpA). The authors concluded that the landfill leach did not cause groundwater contamination, demonstrating a low risk for human health [104]. In other spots, like Hubei, China, mutable concentrations of 12 diverse PFASs have been sensed in the Qing River, with concentrations peaking through summer to the extent of 39–207 ng/L [105]. Another study was carried out on the river–lake system on the Yangtze River in Jiangxi Province, and the occurrence of eleven PFAS types in the surface waters was detected. This prevalence seemingly originated from the municipal wastewater treatment plants (WWTPs). High levels of ΣPFAs in the surface waters of the Nanchang City urban area (146–586 ng/L) and the Jiujiang section of the Yangtze River (46–157.6 ng/L) were detected, with the pronounced occurrence of PFBS and PFOA [106]. A quantitative analysis of C3–C14 PFASs in marine and fluvial sediment samples of fluvoxamine from the coastal areas of the East China Sea was carried out by Yan et al. [107]. The average ΣPFA level was 9 g/g dw, with the highest occurrences shown by PFOS, PFHpA, and PFOA [107]. Yao et al. [108] conducted a local-scale examination of two industrial towns in North China for the evaluation of the PFAS concentrations in the surface rivers and nearby groundwater. In all four rivers investigated in the two cities, the Perfluoroalkyl carboxylic acid (PFCA) class caused over 70% of the detected PFASs. The leading PFCA was PFOA, ranging between 8.6 and 20 ng/L in Tianjin and 6.37–26 ng/L in Weifang, respectively. Samples from the Dagu Drainage Canal (Dagu) in Tianjin showed the uppermost concentration. Short-chain PFASs (C4–C6) were detected at a comparable level to longer-chain PFASs (>C6), with PFBA found to be leading in the short-chain equivalents. This designates an association between the increasing input of short-chain PFASs and industrial discharges or wastewater treatment plant effluent, which could be due to the switching of manufacturing to short-chain products [108].

In the Bohai Sea, Kwok et al. [109] investigated the occurrence of short-chain and long-chain PFASs at the low-water and high-water phases of surface water. The overall concentration of PFASs in seawater was either undetectable or at 99 ng/L. PFOA was the predominant analyte in the water samples, with high levels of PFBS, PFHxS, and PFOS detected, unveiling chronological differences in the water samples. This fact indicated that the differences in seasonal activity characterized the sources of PFAS emissions into the Bohai Sea. In 2017, Chen et al. assessed the PFAS levels in coastal wastewater and river water in the Bohai Sea. In the river water samples, PFBS, PFOA, and PFOS were the principal detected compounds, with ΣPFAs ranging from 13 to 70 ng/L, indicating the existence of sites with high levels of contamination. On the other hand, PFOA was the most signified substance in coastal wastewater, wherein ΣPFAs levels ranged between 16.7 and 7522 ng/L and 13–319 ng/L, respectively. The authors indicated the effect of riverine factors which play a key role in PFAS pollution in the Bohai Sea, with the release of coastal wastewater leading to a reduced effect [110].

In the Xiamen Sea area (China), bioconcentration (BCF) ranged from 6400 to 9700 L/kg to 3300–8000 L/kg for PFOA and PFOS, respectively [111], reflecting the quantity of PFAAs in diverse trophic levels of aquatic animals. In the wild crucian carp, collected from the Yubei River (China), the log 10BAF was 3 (in muscle) and 4 (in blood) [112]. Cui et al. [113] analyzed 35 different PFASs in *Ruditapes philippinarum* and confirmed that the BAFs were associated with C chain length.

In the Xiamen Sea, concentrations ranged between 1.6 and 4.6, 1.4–3.6 and 8–13 ng/g, for PFOA, PFOS, and ΣPFASs, respectively, in kelp algae [111]. The aquatic plants *Salvinia natans Ceratophyllum demersum* L. and *Hydrocharis dubia* (Bl.), collected from Baiyangdian Lake (China), presented high levels of ΣPFAs (≈19.2 ng/g), with marked occurrences of PFOA (≈10.4 ng/g) and PFNA (≈20.1 ng/g). However, PFHpA, PFBS, and PFHxS were not detected [114]. On the Jiaozhou Bay coast, 35 PFASs were detected in *Ruditapes philippinarum* samples, found to be equal to 15–27 µg/kg [113].

23 PFCs were detected in mollusks from a semi-closed basin of the Bohai Sea, displaying a variation between aquaculture sites, with the PFOA being the most detectable (87% of the ΣPFASs), followed by PFNA and PFOS [115]. Of the mollusks sampled in various mussel farms, the highest levels of contamination were exhibited in samples taken near industrial areas touched by anthropogenic pollution.

Li et al. [116] analyzed 21 PFAs in vegetables and fruits collected from agricultural parks in China received from the CFCIP. The ΣPFAS levels were 11.5 and 10.5 ng/g in vegetables and fruits, respectively. In terms of detection frequency and concentrations, PFOA and PFBA were the dominant classes of PFASs. In this study, the PFOA abundance was assessed at 35.5% in the vegetable and fruit samples.

The predominant presence of PFBA in agricultural products might be clarified by the high transfer factor of PFBA from the nutritional matrix to the aerial tissues of plants [117]. Long-chained PFCA was occasionally noticed in a few selection sites with a DF of less than 7%. Qian et al. [118] indicated that long-chained PFCA might accumulate in the root part. In addition, the simultaneous bioaccumulation of PFBA and PFOA was found in melons, solanaceous species, and pears. Moreover, grapes and leafy vegetables exhibited the bioaccumulation of PFOA and PFBA at high levels.

In a previous study conducted by these authors in 2017, Liu et al. revealed a linear positive link between the prevalence of PFCA in agricultural land soil near the Changshu fluorine-chemical industrial park (CFCIP) and PFCA bioaccumulation in maize grains and wheat. These authors noticed that the chief PFASs found in agricultural soils, groundwater, and crops come from the effluent discharges from the CFCIP. Despite the dominance of PFOA in the soil matrix, PFBA presented the maximum levels in multiple crops due to bioaccumulation preferences [119,120]. A few investigations were conducted into vegetables sold at supermarkets and retail stores. A detection frequency of PFBS of 12.5% of the time with a mean concentration of 0.027 ng/g was shown in a supermarket survey in Beijing [99]. That is why tracking PFAS exposure and loading at the diverse steps of the supply chain, along with the improvement of the associated traceability system, is imperative.

Bao et al. [100] reported that ΣPFAs ranged from 63 to 108 ng/g in home-produced eggs. This fluctuation depended on the remoteness (between 0.2 and 1.0 km) from the FIP in China. The same findings were reported by Zafeiraki et al. [121]. These authors concluded that the home-produced eggs were more adulterated than the commercially produced eggs. This is probably due to the controlled feed and environment of neighboring hens for large-scale egg production. Home-grown chicks are more prone to feed and roam in zones that may have been subject to outside contamination.

### 3.2. European Countries

Zafeiraki et al. [122] analyzed PFAs in fish samples, bivalves, crustaceans, and eels captured from Dutch waters or purchased from markets. ΣPFAS levels were highest in eels and shrimp collected from rivers and lakes and the Dutch coast, respectively. The majority of the studied farmed fish had ΣPFASs ranging between 0.06 and 1.5 ng/g. Geographically, the levels in marine fish from the northern North Sea were inferior to those registered in the central and southern North Sea (e.g., flatfish and cod). Regarding eels, no considerable geographical changes were established. The contamination order was comparable in all species, and PFOS and other long-chain PFASs were commonly detected, whereas short-chain PFASs were infrequently detected. Remarkably, a major part of the detected PFOS concentrations in eels (≈93%) and one shrimp sample surpassed the EU Environmental Quality Standard (EQS) for surface water of 9.1 μg/kg. In their investigation, Zafeiraki et al. [122] detected PFOS concentrations at a range of 3.3–67 ng/g, and these findings were reliable when compared with previous studies on eels collected from the Netherlands and other European countries. According to Kwadijk et al. [123], PFOS levels in eels from Dutch rivers ranged from 7 to 58 ng/g in eel muscle. Comparable PFOS concentrations were also reported in eels collected from the Mohne River in Germany (37–83 ng/g) [124] and from the Loire estuary in France (18–39 ng/g) [125]. In contrast, lower PFOS levels were found in eel muscle tissues in Italy (0.3–2.48 ng/g) [126] and Spain (highest 21.6 ng/g ww) [127]. On the other hand, low ΣPFAS concentrations and PFOS levels have been examined previously in mussels from Spain [128], France [129], Greece [130], Denmark [131], and the Mediterranean Sea [132]. In a study conducted by Schmidt et al. [133], four PFASs were detected in the Rhone River from 2017 to 2018. In this investigation, there was a high ΣPFAS range, from 13 to 200 ng/L and high levels of PFHxA (8–193 ng/L) and PFOS, beyond the annual average the EQS in more than 80% of the cases. In the study of Squadrone et al. [134], a substantial correlation between weight and PFOS levels was found in European perch (*Perca fluviatilis* L.) from Lake Varese, Italy.

### 3.3. USA and Australia

Houtz et al. [135] analyzed groundwater samples from Ellsworth Air Force Base in South Dakota, -Piedmont- USA. The middle levels of PFOS and PFOA were 19,000 and 26,000 ng/L, respectively. In addition, higher levels were detected for PFHxS and PFHxA, at 71,000 and 36,000 ng/L, respectively. Braunig et al. [136] examined several biological and environmental matrices sampled from Oakey (Australia). PFOS (4300 ng/L) was the most abundant compound, followed by PFHxS (2300 ng/L), detecting higher concentrations in the groundwater. PFAAs were detected in over 50% of the water samples, and PFOA, PFPA, PFHxA, and PFBS were found at levels between 120 and 600 ng/L. Allinson et al. [137] elucidated 18 PFASs, including PFBS, PFOA, PFBA, PFHxS, and PFOS at 7, 8.5, 11, 42, 75 ng/L, respectively, by examination of the occurrence of common PFASs in surface waters from seven estuaries and creeks.

One of the important advantages of mass spectrometry is its versatility, as well as its ability to be employed for an extensive range of samples, including biological and environmental samples and food products. Examining PFAS mixture complexes with fast analysis times, mass spectrometry presents intense accuracy, specificity, and sensitivity. With the progression of technology, it can still be further developed, making it an important instrument for PFAS researchers. In addition, the orbitraps tool is the most multipurpose mass spectrometer, while triple-quadrupole spectrometers are employed for the targeted detection of several PFASs. Relatively, orbitraps generate the highest peak determination and mass accuracy and offer more mass spectrometric details for the analytes of concern [138]. An additional advantage of orbitrap and triple-quadrupole mass analyzers is their ability to perceive PFAS analyte breakdown. In comparison to linear ion traps, the employment of triple mass spectrometers is inexpensive and more thoughtful. Additionally, nano-liquid chromatography has been recognized to be advantageous by virtue of its inferior consumption of mobile phase solvents, diminishing the costs for both solvents and waste. Along these lines, the quantitative capability of employing matrix-assisted laser desorption/ionization mass spectrometry (MALDI-MS) has been acknowledged to be × 100 faster in comparison to liquid chromatography–tandem mass spectrometry (LC–MS/MS) with lower sample volumes and no organic solvent usage.

While these tools report benefits over conventional MS/MS techniques, they usually have characteristic disadvantages, which will probably stop their extensive implementation in PFAS analysis. Moreover, owing to the reduced sample volumes employed in micro-sampling, MALDI-MS, and nano-liquid chromatography treatment, their quantification ability for PFASs at very low circulatory levels is, likewise, imperfect. In addition, the absence of information on critical method-development phases like internal standards is a limitation that restricts the ability of other researchers to repeat the same work. Therefore, due to the limited number of applications using these advanced technologies up to the present time, which would play a part in the scope of this study, it is problematic to attempt to achieve reproducible and vigorous assessments. Future criticisms that comprise these skills will deliver critical information on specialist applications for PFAS quantification.

Consequently, a summary of the PFASs, their corresponding classes, and the levels found in various food products sampled in different countries is described in Table S1 [99,100,102,111,113,119,121,122,139,140,141,142,143,144,145,146,147,148,149,150,151,152] as a Supplementary File, where PFAS concentrations in food and dietary intake in several countries are shown.

### 3.4. PFASs in Drinking Water and Food: Risks, Mitigation, and Regulatory Needs

PFASs and PFOS constitute a category of synthetic chemicals extensively utilized across multiple industries for their water- and grease-repellent characteristics [153]. Nonetheless, they exhibit persistence in both the environment and the human body, resulting in significant apprehensions regarding their presence in potable water [154]. The details encapsulated in Table 2 delineate the principal risks linked to PFAS chemical contamination in drinking water, water treatment, and food products [155]. The necessity for effective mitigation strategies and the significance of rigorous regulatory measures to safeguard public health is critical [96].

Due to their persistence, bioaccumulation potential, and related health risks, PFAS contamination in drinking water poses a substantial public health challenge [94,157]. Although initiatives to regulate and alleviate PFAS contamination are in progress, enhancing these efforts and allocating resources to advanced technologies and research is essential [96]. Guaranteeing safe drinking water is a fundamental right, and mitigating PFAS contamination is critical to realizing this objective for all communities [167].

Chemical contaminants in food pose a significant and escalating risk to human health. The enduring presence of pollutants such as PFASs, heavy metals, and endocrine disruptors, coupled with inadequate regulatory frameworks in certain areas, complicates the resolution of this issue [168,169,170]. A global initiative is necessary to safeguard consumers, encompassing enhanced regulations, technological innovations, and public awareness campaigns [171,172]. By reducing exposure to hazardous chemicals and implementing more sustainable agricultural practices, we can mitigate the health risks associated with chemical contaminants and progress toward a safer and more sustainable future in food production [159,173,174].

Advancements in food technology, including enhanced detection methods for chemical contaminants and novel processing techniques, can mitigate contamination risks [175,176]. Enhanced filtration systems for irrigation water and innovative agricultural practices that minimize pesticide usage can reduce contaminant levels in crops [177].

Public awareness is essential. Consumers should be informed of the risks associated with certain food packaging and encouraged to limit their exposure by choosing safer alternatives [155]. Furthermore, focusing more on home-cooked meals with fresh ingredients helps to reduce exposure to toxins found in processed and fast foods [173].

Governments and international organizations must collaborate to align safety standards and regulations, ensuring uniform protection across borders, especially for vulnerable populations in developing nations [178,179].

It is essential to prioritize investment in research to create safer alternatives to hazardous food packaging materials, including PFASs and BPA [177,180].

The augmented monitoring and testing of food products throughout the supply chain can facilitate early the detection of contamination and avert the distribution of hazardous products to consumers [178,181].

Enhanced public education regarding the hazards of chemical contaminants in food and methods for their avoidance should constitute a comprehensive strategy to mitigate consumer risk [181,182].

In conclusion, the pervasive presence of PFAS contamination in food and drinking water presents a significant public health challenge. These chemicals’ persistence and bioaccumulation potential underscore the urgent need for stringent regulations, continuous monitoring, and innovative remediation strategies. By addressing PFAS contamination, we can protect ecosystems and ensure safer consumption of food and water for future generations [183].

### 3.5. Chemical Contaminants in Food and Their Impact on Health and Safety

As we pivot from the issue of PFAS contamination, it is essential to broaden our focus to the myriad chemicals found in our food. From pesticides and additives to packaging materials, the journey from farm to table involves exposure to various substances. Understanding their impacts, regulatory frameworks, and safety measures is crucial for safeguarding public health and maintaining food integrity.

Chemically contaminated foods have become a global health issue, presenting risks from mild gastrointestinal ailments to serious long-term conditions, such as cancer and developmental disorders [178]. The escalation of industrialization, globalization, and evolving agricultural methods has intensified these risks, rendering chemical contaminants a pressing concern for regulators and the food sector [172,184]. This study offers a comprehensive analysis of the principal findings from various studies and articles regarding chemical contaminants in food, encompassing the sources of contamination, its effects on human health, and the requisite measures for prevention [185]. Significant attention is directed towards per- and PFASs, BPA, and other harmful compounds that leach from packaging into food [168,186,187].

Despite initiatives to eliminate long-chain PFASs like PFOA and PFOS, shorter-chain PFASs have emerged as substitutes in numerous products [183]. Research indicates that although these shorter-chain PFASs are less bio-accumulative, they still present considerable risks to human health, including hepatic damage, immune system suppression, and developmental disorders [169,186].

Furthermore, chemical contaminants in food pose a significant and escalating risk to human health. The enduring presence of pollutants such as PFASs, heavy metals, and endocrine disruptors, coupled with inadequate regulatory frameworks in certain areas, complicates the resolution of this issue [172,185,187]. A global initiative is necessary to safeguard consumers, encompassing enhanced regulations, technological innovations, and public awareness campaigns [168,171]. By reducing exposure to hazardous chemicals and implementing more sustainable agricultural practices, we can mitigate the health risks associated with chemical contaminants and progress toward a safer and more sustainable future in food production [173,174].

Several studies have identified safer and more environmentally friendly alternatives to PFASs. For example, the US Environmental Protection Agency (EPA) has highlighted strategic research areas, including the development of PFAS alternatives [188]. Additionally, an article by Battelle discusses the pressure on companies to remove PFAS chemicals from their products and explores potential alternatives [189]. Another study by the Food Packaging Forum maps PFAS applications and identifies suitable alternatives [188].

**Figure 3:**
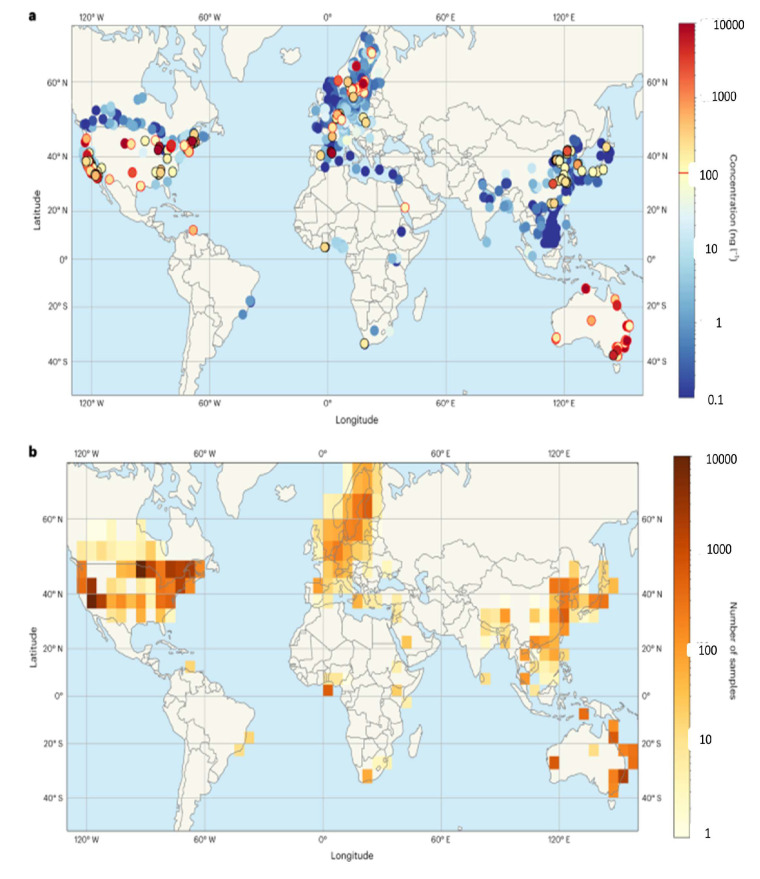
Global map of PFASs concentration in water. (**a**) Sum of concentration of 20 PFASs subject to EU guidance in surface water, groundwater, and drinking water samples. Those above the EU drinking water limit of 100 ng L^−1^ (marked red on the scale bar) are circled in red (for known contamination sources (for example, AFFF or non-AFFF)) or black (unknown sources). (**b**) Number of PFAS samples available on a 5° longitude/latitude grid worldwide [190].

A global map of PFAS concentration in water is shown below in Figure 3.

## 4. Regional PFAS Impact and Activities Briefings

The impact and activities surrounding PFASs (per- and polyfluoroalkyl substances) vary significantly across regions worldwide. Each briefing in this section serves as a standalone report, meticulously crafted to raise awareness, educate the public, and address the multifaceted elements of the global PFAS crisis within the context of the specific region. By delving into regional nuances, these briefings aim to give a full grasp of the effects of PFAS contamination on ecosystems, human health, and regulatory landscapes around the globe. The regional briefings below represent the more active countries (developed and developing) in the context of PFAS awareness, regulatory activities, studies, and mitigation/prevention strategies.

### 4.1. Australia Briefing: PFAS Impact and Activities

In Australia, PFAS contamination has been linked to firefighting foams and industrial processes [191]. Recognized as toxic, these chemicals are being phased out globally, including in Australia. PFAS contamination is found in soil, surface water, and groundwater, with most Australians having measurable levels in their blood [192,193].

The Expert Health Panel on PFASs found limited scientific evidence of health effects in humans, but links to elevated cholesterol, kidney issues, specific cancers, and vaccine responses exist. PFAS exposure is also linked to cancer risks, reproductive health concerns, and environmental contamination [191,194,195]. Continuous monitoring and research are essential for understanding long-term health impacts.

The Australian government has adopted a precautionary approach, including health-based guidance values, drinking water guidelines, research initiatives, and biomarker testing [193]. New South Wales Health aids water suppliers in testing for PFASs beyond Defense Force bases and airports. While the US EPA considers PFASs in drinking water unsafe, Australian guidelines set a safe level of 0.07 µg/L, with Victoria monitoring drinking water for contamination [196,197,198,199].

A Melbourne study found PFASs in groundwater near recycled water irrigation sites, raising drinking water concerns. A national study found PFOS and PFOA in about half of the tested samples, highlighting the need for ongoing public health testing [137,191]. No maximum PFAS limits for food have been set by Australian regulators or internationally, but the Food Standards Australia New Zealand (FSANZ) agency has developed non-regulatory “trigger points” for livestock products, seafood, fruits, and vegetables [200].

A study in Australia discovered that perfluorinated alkyl acids (PFAAs) are persistent contaminants in human serum and water treatment systems. PFOS and PFOA were the most detected, with drinking water contributing 2–3% of total exposure, reaching up to 22% and 24%, respectively [201].

PFASs pose significant risks to marine life through bioaccumulation, toxicity, and biomagnification [194]. PFOS was found in eight of nine platypus livers, with health concerns raised [197]. Australian freshwater fish and crustaceans show PFOS concentrations above the Australian trigger value, needing more research on toxicological and reproductive effects [196].

PFOS accumulates in bottlenose dolphin livers, posing health risks [202]. PFOS and PFOA are toxic to sea urchins, mussels, and shrimp, affecting their development and survival [203]. Higher PFAS levels were found in Australian sea lion and fur seal pups near defense bases and airports [204]. PFAS compounds accumulate in marine food webs, raising ecological concerns [205].

PFASs accumulate in livestock, affecting grazing behaviors and water requirements. In Victoria, the impact on animals is low, reducing community exposure through meat production [206].

Australia has guidelines for the handling and disposing of PFASs, but stronger enforcement is needed. No maximum limits for PFASs in food have been set by regulators [207,208,209]. PFAS levels are higher near sewage treatment plants, landfills, and firefighting foam sites. The disposal of PFAS-contaminated waste is permitted only in landfills with specific lining systems [210]. The EPA anticipates more disposal options, with the PFAS National Environmental Management Plan providing guidance [184]. The Waste Management and Resource Recovery Association of Australia has urged the federal government to ban all types of PFASs by 2025 [211].

With reference to microplastics in the ocean, the Great Pacific Garbage Patch (GPGP) is the largest offshore plastic accumulation zone globally, receiving 1.15 to 2.41 million tons of plastic annually from rivers. The GPGP’s plastic mass is around 100,000 tons, with 80% from land-based sources. Microplastics block sunlight for plankton and algae and leach harmful contaminants [212].

In Australia, public support has grown for synthetic biology solutions for bioremediation in waterways [194]. Electrokinetic bioremediation uses microbial survival and enzyme secretion to treat polluted soils. The CRC CARE workshop in 2019 identified research gaps in managing PFAS contamination in Australian soils and groundwater. Phycoremediation using algae, such as *Synechocystis* spp., offers a green, sustainable water treatment alternative [213,214,215,216].

Emerging technologies like electrochemical oxidation and supercritical water oxidation are being explored for PFAS waste remediation. CSIRO has collaborated with industry partners on bioremediation using microbes [217].

Government agencies assess PFAS contamination, particularly at military bases. Monitoring programs track PFAS levels in the environment and populations [218].

Projects include soil excavation, groundwater treatment, and using activated carbon to remove PFASs from water [192].

Health advisories protect communities affected by PFASs, including providing alternative water supplies and dietary advice [191].

Australia faces legal action due to PFAS contamination. Affected communities seek compensation for property-value loss, health impacts, and environmental damage [219,220].

The Australian Senate is investigating PFAS contamination to develop remediation strategies and improve public health protection [221].

Research is crucial for understanding PFASs. Australia participates in international initiatives, and strengthening regulations and monitoring programs is vital for protecting human health and the environment [194,222].

### 4.2. Vietnam Briefing: PFAS Impact and Activities

Vietnam’s shift from a centrally planned to a market economy has raised it from one of the world’s least economically developed countries to a lower-middle-income one. Vietnam is one of the fastest-growing countries in Southeast Asia [223].

The Vietnamese government continues to increase its focus on regulating chemicals, including per- and poly-fluoroalkyl substances (PFASs), often called “forever chemicals”. Though Vietnam has yet to regulate all PFASs, it has made progress in managing persistent organic pollutants (POPs), which include some PFASs like PFOA and PFOS. These efforts align with Vietnam’s obligations under the Stockholm Convention, which it has been a party to since 2004 [224]. PFOS use has been limited since 2010, and the government continues to monitor PFAS pollution in water and seafood [225,226].

Vietnam’s Ministry of Natural Resources and Environment (MONRE) and the Ministry of Industry and Trade (MOIT) have also been urged to strengthen PFAS monitoring and conduct further research on their environmental and health impacts. These studies and the global trend to phase out PFASs in products like firefighting foams will inform future regulations [225,226,227].

Vietnam is developing PFAS handling and disposal guidelines. The current guidelines for chemical management, including hazardous chemicals like PFASs, fall under the Law on Chemicals and the Environmental Protection Law. The MONRE and other regulatory bodies have issued guidelines for safely handling and disposing of POPs, including some PFAS compounds like PFOA and PFOS.

The government is working to compile a PFAS chemical inventory, especially in firefighting foams and industrial uses. This is seen as a necessary step for understanding where PFASs are used and where the highest pollution risks exist.

Vietnam has strong waste management rules, but PFAS disposal methods are still developing. Waste treatment facilities are regulated to prevent environmental releases. To avoid environmental contamination, hazardous waste like PFAS-contaminated materials must be treated in controlled environments. Vietnam must follow international PFOS and similar chemical disposal guidelines as a signatory of the Stockholm Convention. This involves eliminating or reducing the use of these chemicals in products and ensuring proper destruction methods for waste containing these substances [224,227,228].

While Vietnam’s PFAS handling and disposal guidelines are still developing, ongoing legislative amendments and international cooperation are expected to guide future regulatory measures. Compared to the US and Europe, where PFAS issues are more widely reported, public awareness about PFASs in Vietnam is low. In Vietnam, PFAS data are scarce, and most people are unaware of the health and environmental risks [226].

However, there have been efforts to raise awareness, mainly through reports and campaigns from environmental organizations, which have worked on surveys and research in collaboration with international organizations like the International Pollutants Elimination Network (IPEN) [229]. The Vietnamese government has acknowledged PFAS contamination in water and seafood, despite the public’s ignorance. Pollution is not yet part of the country’s mainstream public discourse, despite media coverage and environmental studies [228].

Increased government action on PFAS regulation and monitoring, as well as international collaborations, could raise public awareness of PFAS dangers. Vietnam lacks broad initiatives to develop innovative PFAS bioremediation solutions. The country is interested in wider environmental and pollution control technologies, especially regarding international agreements like the Stockholm Convention, working with global organizations and experts to address POPs like PFASs [226].

Vietnam will adopt or collaborate on global bioremediation techniques due to the growing awareness of PFAS contamination. Vietnam’s environmental strategy now emphasizes monitoring, regulating, and preventing PFASs from spreading in water, food, and industrial sites [226].

Most PFAS bioremediation innovations are in the US, Europe, and Australia, where research is advanced. These initiatives include developing technologies that use microbes or engineered enzymes to break down PFAS compounds, which could be crucial for addressing water and soil contamination in countries like Vietnam in the future [230].

Testing for PFASs in Vietnamese drinking water is not yet a widespread government initiative. PFAS monitoring is limited, and testing is not usually part of public water quality assessments. But studies and reports, especially from environmental organizations, show PFAS contamination in water sources near industrial areas [226]. The government has been advised to enhance PFAS testing, especially in groundwater and near suspected hotspots like industrial areas and places using firefighting foams. The MONRE and MOIT should monitor together [226].

The Vietnamese government has made progress in regulating POPs like PFOS and PFOA in food products under international conventions like the Stockholm Convention. However, PFAS monitoring and rules are limited, especially for drinking water. Vietnam has no PFAS Maximum Residue Limits for food yet. Vietnam’s chemical management laws, including those for PFASs, are still developing [226].

PFAS chemicals are highly persistent and do not break down easily in the environment, leading to their accumulation in water, sediments, and living organisms. Research has shown that PFAS exposure can negatively affect marine ecosystems: PFAS compounds can accumulate in the tissues of marine organisms, especially those higher up the food chain, such as fish and marine mammals. These chemicals accumulate over time, harming predators, including humans, who eat tainted seafood [231]. Studies have demonstrated that PFASs can affect marine life’s reproduction, growth, and immune functions. PFOS and PFOA (common PFAS compounds) exposure has been linked to developmental issues, liver toxicity, and endocrine disruption in fish and amphibians [232]. PFASs in water can disrupt aquatic ecosystems by affecting the health and survival of species at various food web levels. This pollution affects fish and other marine life, such as crustaceans, mollusks, and seabirds that depend on these food sources [233].

PFASs are a long-term threat to marine ecosystems due to their persistence and bioaccumulation. International studies and environmental monitoring programs increasingly recognize the need for better management and remediation strategies to mitigate the harmful effects of PFASs on marine life [233]. As awareness of PFAS risks increases, there may be future developments in setting safety thresholds for food products, particularly as Vietnam aligns more closely with international standards and regulations concerning chemical contaminants in food [226].

PFASs in food packaging and pesticides: Vietnam has no bans or limits on PFASs in food packaging. The country is now regulating PFOS and PFOA under international obligations. Food packaging has been less of a focus than industrial applications and environmental pollution [226].

PFAS use in pesticides is a growing concern in some countries, but little is known about their use or regulation in Vietnam’s agriculture. Vietnam’s chemical management framework is evolving, and while some hazardous industrial chemicals are monitored, PFASs in pesticides are not addressed in the country’s laws. But due to global trends and the growing awareness of PFASs’ environmental and health effects, Vietnam may soon follow other countries in limiting their use in farming, including pesticides [226].

Vietnam is involved in many global initiatives to study and control PFAS pollution. These partnerships enhance Vietnam’s capability to address PFAS challenges by sharing information and resources and working together. Vietnam’s major global partnerships and initiatives especially include the Stockholm Convention on POPs, an international treaty to which Vietnam is a party. It aims to eliminate or restrict the production and use of POPs, including PFOS and PFOA. Additionally, Vietnam is bound by phasing out the production and use of PFASs [234].

Vietnam regularly monitors POPs in the environment and reports the findings to the Convention’s Secretariat. Training and technical help are required to boost national skills to manage and reduce POPs [234].

Vietnam works with international NGOs and networks to address PFAS contamination. Vietnam collaborates with the IPEN to eliminate toxic pollutants like PFASs through surveys, research, and advocacy. This partnership helps Vietnam devise PFAS management and remediation plans [229].

As part of its membership in the IPEN, PanNature engages in projects that monitor PFAS pollution and promote sustainable practices to reduce contamination [226]. ASEAN (Association of Southeast Asian Nations) environmental initiatives on chemical management and pollution control include Vietnam. Regarding ASEAN POPs protocol, this regional agreement complements the Stockholm Convention by addressing POPs within Southeast Asia. Vietnam works with nearby nations to align laws, share ideas, and launch PFAS reduction initiatives. Vietnam participates in regional capacity-building efforts, including training on PFAS detection, management, and remediation procedures [235].

Vietnamese universities and research centers partner with international institutions to advance PFAS studies. Collaborative research initiatives focus on understanding the environmental and health impacts of PFASs, developing innovative remediation technologies, and assessing the effectiveness of regulatory measures [230]. Knowledge Exchange Programs promote the exchange of technical knowledge and expertise between Vietnamese scientists and their international peers, fostering PFAS management innovation [230].

With regard to international organizations’ technical assistance and funding, Vietnam receives global support to improve PFAS management. The United Nations Environment Programme (UNEP) provides technical assistance, funding, and guidance to help Vietnam implement PFAS regulations, conduct environmental assessments, and develop remediation strategies [236]. Organizations such as the World Bank and other funding agencies may fund projects to reduce PFAS pollution, improve waste management systems, and upgrade industrial processes to minimize PFAS emissions [236].

Vietnamese representatives actively attend global conferences and forums on chemical safety and pollution control. Events such as Global Chemical Safety Forums allow Vietnamese policymakers and scientists to share experiences, learn about global PFAS management advancements, and collaborate on international initiatives. Attending specialized workshops helps Vietnam stay current on PFAS research, technology, and regulations. Vietnam adopts international PFAS management best practices through these collaborations. It tries to integrate global norms and guidelines into national legislation to regulate PFAS use and emissions effectively [234]. It uses global expertise to create and execute campaigns that inform the public and industries about PFAS risks and the need for responsible chemical management [229]. Vietnam is expanding global partnerships to tackle PFAS issues. Among its future plans are a more comprehensive PFAS monitoring system with global partners [226], the investment in new technologies developed through global research partnerships to effectively remediate PFAS-contaminated sites [222], and the creation of strong PFAS regulations that meet global standards [236].

Vietnam’s PFAS research and management efforts depend on its participation in international collaborations. Vietnam is improving its ability to reduce the health and environmental effects of PFAS contamination by using global expertise, resources, and strategies. These partnerships help the nation and the world to combat persistent organic pollutants.

PFAS contamination is a growing global issue in landfill management, including in Vietnam. PFAS compounds are often found in landfill leachate, which can carry these toxic chemicals into groundwater and surrounding ecosystems [231]. Landfills can be a major source of PFAS contamination since many PFAS-containing consumer products—like food packaging, textiles, and non-stick products—end up there. PFASs can leach into the environment as these products break down [230].

Effective landfill management strategies for PFASs include containment, the treatment of leachate, and limiting the types of PFAS-containing products that enter landfills. As Vietnam aligns its environmental policies with international standards, the awareness of PFAS contamination grows, and it becomes beneficial to do so, the country will likely focus more on this issue [230]. PFASs in water or soil near landfills or industrial zones could threaten crop safety in Vietnam, where agriculture is vital to the economy [231]. Since PFASs are resistant to degradation, they can remain in agricultural environments for extended periods, impacting the quality and safety of food products [232]. Despite a lack of widespread regulation or testing for PFASs in Vietnamese agriculture, international concern and new research suggest that this issue will require more attention in the future [233].

### 4.3. Canada Briefing: PFAS Impact and Activities

Canada regulates PFASs under the Chemicals Management Plan (CMP), which evaluates and manages substances posing risks to health or the environment [237]. Environment and Climate Change Canada (ECCC) guides the managing and disposing of PFASs, including regulatory requirements under the CEPA [238]. Canadian universities and environmental agencies are involved in research, with support from Natural Resources Canada (NRCan) [239]. Municipalities follow Health Canada, which sets the health guidelines for PFASs in drinking water [240].

Canada has not yet established specific MRLs for PFASs in food products. The public can track the latest developments under the Pesticide Residue Program from the Canadian Institute for Food Safety [241]. Canada restricts certain PFASs under the Canadian Environmental Protection Act (CEPA). For example, the Prohibition of Certain Toxic Substances Regulations list specific PFASs (like perfluorooctanoic acid (PFOA) and perfluorooctane sulfonate (PFOS)) as prohibited substances, meaning their use in products, including food packaging, is restricted [242]. Under the Pest Control Products Act, the Pest Management Regulatory Agency (PMRA) oversees the regulation of pesticides. PFASs are considered under this framework when evaluating pesticides for registration, and certain PFAS chemicals are restricted from use in agricultural products due to their toxicity and persistence in the environment [243].

Canada regulates PFAS discharge into waterways through several legal frameworks, including the CEPA and the Fisheries Act. The Pollution Prevention Provisions of the Fisheries Act make it illegal to deposit harmful substances, including PFASs, into water that could affect fish or fish habitats [244]. In addition, the Wastewater Systems Effluent Regulations (WSERs) establish national effluent quality standards for pollutants discharged into waterways, including toxic substances like PFASs [245].

Canada actively participates in international efforts such as the Stockholm Convention on persistent organic pollutants (POPs), which seeks to eliminate or restrict the production and use of persistent organic pollutants like PFASs [244]. Canada has committed to phasing out specific PFASs under this convention [246]. Canada also collaborates with the Organization for Economic Co-operation and Development (OECD), particularly within the OECD’s PFAS Working Group, which facilitates international cooperation on PFAS management, research, and data sharing [247].

International collaboration has positively impacted Canada’s approach to PFASs. For example, Canada’s commitment to the Global Monitoring Plan under the Stockholm Convention has led to improvements in environmental monitoring and the regulation of PFASs. Additionally, knowledge sharing with the European Union and the United States has influenced Canada’s regulatory policies and risk assessments for PFASs. This international cooperation has led to a better scientific understanding and more-effective national actions in reducing PFAS exposure [248].

Through several government and academic institutes, Canada is aggressively researching the long-term health effects of per- and polyfluoroalkyl substance (PFAS) exposure. With an emphasis on PFASs’ impacts on human health, particularly its connections to cancer, thyroid disorders, and immune system disruption, Health Canada has carried out many risk evaluations on the chemicals [249]. Studies on PFASs are also funded by the Canadian Institutes of Health Research (CIHRs), which also look at how they affect vulnerable groups like children and pregnant women [250]. Furthermore, because higher amounts of PFASs have been found in wildlife and food sources in Arctic populations, the Northern Contaminants Program (NCP) is looking into the consequences of PFASs there [251].

PFASs are increasingly recognized as a critical public health issue in Canada due to their widespread environmental presence, persistence, and potential for adverse health effects [252]. Health concerns are heightened in communities where PFAS contamination has been identified (e.g., near military bases, firefighting training sites, or industrial facilities), and governments are taking steps to mitigate exposure [253]. Their efforts include stricter regulations, improved water testing, and public health advisories to reduce PFAS risks [246].

### 4.4. Europe Briefing: PFAS Impact and Activities

PFASs have been detected in food and drinking water across Europe [96]. A study found that over 99% of bottled water samples from 15 countries contained PFASs [254]. These chemicals are persistent in the environment and can accumulate in the bodies of living organisms [255].

The European Chemicals Agency (ECHA) proposed a ban on approximately 10,000 PFASs in February 2023 [256]. This proposal aims to restrict the production, use, and sale of PFASs in consumer products [256]. The ban is part of the EU’s REACH regulation, which seeks to protect human health and the environment from hazardous chemicals [256].

Public awareness campaigns, such as the Forever Pollution Project, have highlighted the widespread contamination by PFASs across Europe [254]. These campaigns aim to inform the public about the risks associated with PFASs and encourage regulatory action [254].

Several innovative projects are underway to address PFAS contamination in Europe [257]. These projects focus on PFASs’ detection, distribution, treatment, and holistic strategies to reduce their environmental impact. Techniques such as bioremediation, chemical oxidation, and advanced oxidation processes are being explored [258].

Monitoring PFAS levels in drinking water is crucial for assessing exposure and health risks [25]. The European Environment Agency (EEA) has highlighted the need for the systematic mapping and monitoring of potentially polluted sites [259]. National monitoring activities have detected PFASs in the environment across Europe [259].

The Maximum Contaminant Level (MCL) for PFASs in drinking water varies across Europe. The European Union has set guidelines to limit PFAS concentrations in drinking water to protect public health [256].

Several lawsuits have been filed in Europe concerning PFAS contamination. These lawsuits often involve claims of environmental damage and health impacts due to PFAS exposure [256].

The ECHA and other regulatory bodies have issued guidelines and recommendations for managing PFAS contamination. These guidelines aim to reduce PFAS use, improve monitoring, and promote remediation efforts [256].

PFAS exposure has been linked to a range of health issues, including increased cholesterol levels, changes in liver enzymes, thyroid disease, decreased vaccine response in children, and an increased risk of kidney and testicular cancers [25]. The toll on human health is significant, with teenagers in Europe facing health risks from exposure to PFASs [259].

The cost of PFAS remediation in Europe is substantial. Estimates suggest that the societal costs due to harm to human health and remediation efforts are tens of billions of EUR annually [256]. Professor Hans Peter Arp estimates the cleaning costs to be EUR 238 billion in the EU alone, with global costs extrapolated to EUR 16 trillion per year [260].

The mismanagement of landfills in Europe has led to significant environmental and public health concerns. Unauthorized landfill sites can generate emissions, unpleasant odors, and contaminate nearby soil and watercourses [261]. Proper management and regulation are essential to mitigate these risks.

Plastic recycling in Europe faces several challenges. Only about 30% of plastic waste is collected for recycling, while 43% is incinerated, and 25% is still landfilled. A substantial proportion of plastics end up in the sea, posing environmental threats [189,262,263,264,265,266]. Improving recycling infrastructure and reducing waste exports are crucial steps toward addressing these issues [267].

Food contact packaging in Europe is regulated to ensure safety. However, mismanagement and non-compliance with regulations can lead to PFAS contamination; ensuring that all food contact materials comply with EU regulations is essential to protect public health and food quality [268].

The widespread contamination of PFASs poses a significant challenge to achieving the Sustainable Development Goals (SDGs) of 2030. Efforts to remediate PFASs and reduce their use are crucial for protecting human health and the environment. The forecast for PFAS remediation globally involves significant investment and regulatory action to mitigate their impact [269].

PFAS contamination is a significant environmental and public health issue in Europe. Efforts to ban, regulate, and remediate PFASs are ongoing, with public awareness campaigns playing a crucial role in driving regulatory action. Continued research and monitoring are essential to mitigate the impact of PFASs on human health and the environment.

### 4.5. USA: PFAS Impact and Activities

Per- and polyfluoroalkyl substances (PFASs) have emerged as a significant environmental and public health concern in the United States. These “forever chemicals” are known for their persistence in the environment and potential to cause adverse health effects. The federal and state governments have been actively working to address PFAS contamination through various legislative and regulatory measures [96,270].

PFASs have been detected in water supplies, soil, and food products nationwide, leading to widespread exposure. Studies have linked PFAS exposure to various health issues, including cancer, liver damage, and immune system effects [271]. The Environmental Protection Agency (EPA) has established the first ever national drinking water standard for PFASs to protect communities from these harmful chemicals [156].

The Biden–Harris administration has taken several bold actions to tackle the PFAS crisis. In April 2024, the EPA designated two widely used PFASs—PFOA and PFOS—as hazardous substances under the Comprehensive Environmental Response, Compensation, and Liability Act (CERCLA), also known as the Superfund. This designation aims to improve transparency and accountability in cleaning up PFAS contamination [170,272]. Additionally, the EPA has issued a national drinking water standard and provided funding to help states and territories implement PFAS testing and treatment [156].

Recent advancements in PFAS bioremediation have shown promise for scalable solutions. One innovative approach involves using plant-based materials combined with microbial fungi to adsorb and degrade PFASs. This method, known as Renewable Artificial Plant for In Situ Microbial Environmental Remediation (RAPIMER), utilizes corn stover to create a porous framework that supports fungal growth and PFAS degradation [271].

To address PFAS contamination in the food industry and agriculture sectors, several capacity-building programs have been initiated [273]. The USDA has developed a roadmap to tackle PFASs on farmland, focusing on detecting contamination, developing tools to prevent harm, and promoting scientific exchange among farmers, scientists, and stakeholders. These programs aim to reduce PFAS risks in food crop production and enhance sustainable farming practices [274]. Numerous public awareness guidance campaigns have been promoted, such as the NRDC October 2024 Fact Sheet, “Toxic Drinking Water: Addressing the PFAS Contamination Crisis” [275].

PFAS contamination in landfills is a significant environmental concern. PFAS-containing products, such as clothing, carpets, bedding, and food packaging, can release PFASs into landfill leachate, contaminating soil and groundwater. Some landfills divert leachate for treatment at wastewater treatment plants, but the challenge remains to effectively manage and mitigate PFAS contamination [276,277].

PFAS contamination in waterways is a critical issue. PFASs can enter waterways through various pathways, including industrial discharge, landfill leachate, and runoff from contaminated agricultural lands. The EPA has been awarded research grants to study the impact of PFASs on waterways and develop strategies to reduce contamination. Efforts are underway to improve water treatment technologies and prevent PFASs from entering water sources [278,279,280,281,282].

The efforts to address PFAS contamination in the USA reflect a growing recognition of the need for stringent regulations, the implementation of HACCP-based food safety systems [283], and proactive measures to protect public health and the environment. By implementing comprehensive legislation and regulatory actions, the government aims to mitigate the impact of PFASs and ensure safer living conditions for all Americans [284].

### 4.6. South America: PFAS Impact and Activities

South American countries have been implementing various regulatory policies to address PFAS contamination. For example, Brazil has established guidelines for PFAS levels in drinking water and soil, while Argentina has introduced restrictions on the use of PFASs in industrial processes. These policies are designed to reduce PFAS exposure and mitigate environmental impacts [285]. Additionally, Peru has set limits on PFAS concentrations in wastewater discharges from industrial facilities, a critical measure to prevent the contamination of water bodies and soils. Other countries in the region are considering similar regulations to effectively control PFAS pollution.

Several South American nations have enacted bans on particular PFAS compounds. For instance, Chile has prohibited the use of PFOS and PFOA in firefighting foams, while Peru has restricted the use of PFASs in consumer products such as non-stick cookware and stain-resistant fabrics. In Uruguay, the government has taken proactive steps by banning the import and production of certain PFAS-containing products. These bans are part of broader efforts to phase out harmful PFAS chemicals and promote safer, more environmentally friendly alternatives. As the awareness of PFAS risks grows, other countries in the region are expected to follow suit [286].

South American countries are actively collaborating with global and regional agencies to tackle PFAS pollution. Key partners include the Inter-American Development Bank (IDB) [287] and the United Nations Environment Programme (UNEP) [288], which work together to share best practices, conduct joint research, and implement regional initiatives [287]. In addition, South American nations adhere to guidance from the Stockholm Convention [289] on persistent organic pollutants (POPs), which regulates PFOS, PFOA, and other PFAS compounds. Regional organizations, such as Mercado Común del Sur {Mercosur} [287], have also been instrumental in coordinating efforts to address PFAS contamination across member countries.

Several scientific studies in South America have focused on the health risks associated with PFAS exposure and on developing innovative bioremediation methods. Research indicates that PFASs can accumulate in humans, animals, fish, and plants, potentially leading to endocrine disruption, immune system dysfunction, and certain cancers [290]. In the area of bioremediation, promising approaches are under investigation. One such method, known as the Renewable Artificial Plant for In Situ Microbial Environmental Remediation (RAPIMER), utilizes plant-based materials combined with microbial fungi to break down PFASs. This technique employs corn stover to create a porous framework that adsorbs PFASs and supplies nutrients for the fungi to degrade the chemicals [291].

Health risk studies in South America have identified clear associations between PFAS exposure and adverse health effects. For instance, research in Brazil has shown that PFAS exposure may lead to increased cholesterol levels, altered liver enzymes, and potential impacts on the immune system [292]. Similarly, a study in Argentina highlighted the risks of endocrine disruption and reproductive issues in wildlife due to PFAS exposure [293]. These findings underscore the importance of continued research and regulation to mitigate PFAS exposure and protect public health in the region.

Chemical waste dumping remains a significant environmental challenge in South America. The Riachuelo River in Buenos Aires, Argentina, is widely considered one of the most polluted waterways in the region, with heavy metals, chemicals, and organic waste being discharged into its waters [294]. Although the Argentinian government has launched an ambitious cleanup project, significant challenges remain in addressing the extensive contamination. In another instance, the Sarandí stream near Buenos Aires turned crimson red due to suspected industrial chemical dumping, sparking fears of toxic leaks. Residents have reported recurring episodes of unusual water discoloration and an oily surface since the 1990s, with many filing complaints against local businesses [295].

Efforts to improve landfill and chemical waste management are also underway in South America. Collaborations between the UNEP [288] and the Global Environment Facility (GEF) [296] are strengthening national capacities for the safe management and elimination of hazardous chemicals and wastes. For example, Argentina has developed a comprehensive waste management strategy that includes the establishment of controlled landfills and the promotion of recycling, along with Extended Producer Responsibility (EPR) [295] systems that hold producers accountable for the lifecycle of their products. In Brazil, initiatives are being implemented to improve waste collection and disposal—particularly in urban areas—through regulations that encourage the separation of recyclable materials and the use of environmentally friendly disposal methods. These efforts aim to reduce landfill waste and foster a circular economy [297].

South America faces a range of environmental challenges, including deforestation, pollution, and climate change. The region’s rich biodiversity is threatened by habitat destruction and the overexploitation of natural resources. Rapid urbanization and industrialization have further contributed to increased pollution levels in air, water, and soil [295].

One of the main challenges is the effective enforcement of environmental regulations. Despite the existence of policies, many countries struggle with limited resources, corruption, and a lack of political will, which often results in ongoing environmental degradation. Additionally, the scarcity of comprehensive data and research makes it difficult to fully understand and address these environmental issues [286].

The future outlook for South America is marked by both significant challenges and promising opportunities. Growing awareness and a stronger commitment to sustainability are driving collaborative efforts among governments, non-governmental organizations NGOs, and international organizations. Investments in renewable energy, sustainable agriculture, and conservation programs are expected to play a crucial role in shaping the region’s environmental future. Moreover, advancements in bioremediation and pollution control technologies will be essential in mitigating the impacts of environmental contamination [286].

### 4.7. Africa: PFAS Impact and Activities

Several African countries have begun implementing regulatory policies to address PFAS contamination. For instance, South Africa has established guidelines for acceptable PFAS levels in drinking water and soil, while Kenya has introduced restrictions on the use of PFASs in industrial processes. These measures are designed to reduce exposure and mitigate environmental impacts. Additionally, Nigeria has set limits on PFAS concentrations in wastewater discharges from industrial facilities, a critical step in preventing the contamination of water bodies and soils. Other countries in the region are considering similar regulations to control PFAS pollution effectively [288,296,298].

Several nations across Africa have enacted bans on particular PFAS compounds. For example, South Africa has prohibited the use of PFOS and PFOA in firefighting foams, while Kenya has restricted PFASs in consumer products, such as non-stick cookware and stain-resistant fabrics. In Uganda, the government has banned both the import and production of certain PFAS-containing products. These proactive steps aim to phase out harmful chemicals and promote safer, environmentally friendly alternatives. As awareness of PFAS risks grows, similar bans are expected to be implemented in other African countries [299,300,301,302].

African countries are actively partnering with global and regional organizations to combat PFAS pollution. Key collaborations include work with the UNEP [288] and the GEF [296] which focuses on sharing best practices, joint research initiatives, and the implementation of regional action plans. In addition, guidance from the Stockholm Convention on POPs, which regulates PFOS, PFOA, and other PFAS compounds, is being followed [296]. Regional bodies, such as the African Union (AU) [303], also play a significant role in coordinating these efforts across member states [304].

Recent scientific studies conducted in Africa have highlighted the potential health risks associated with PFAS exposure as well as promising bioremediation strategies. Research in South Africa, for example, indicates that PFASs can accumulate in humans, animals, fish, and plants, potentially causing endocrine disruption, immune system dysfunction, and an increased risk of certain cancers [298,300,305]. In the realm of bioremediation, innovative approaches are under investigation. One promising technique, known as the Renewable Artificial Plant for In Situ Microbial Environmental Remediation (RAPIMER), employs plant-based materials combined with microbial fungi to break down PFASs [288,296,298]. This method uses corn stover to create a porous matrix that both adsorbs PFASs and supplies nutrients to the degrading fungi [306].

Health risk assessments in Africa have identified clear associations between PFAS exposure and adverse health effects. For instance, a study in South Africa found that exposure to PFASs may lead to elevated cholesterol levels, alterations in liver enzyme activity, and potential immune system impacts. Similarly, research in Uganda has underscored the risks of endocrine disruption and reproductive issues in wildlife due to PFAS exposure [298,300,305]. These findings reinforce the need for ongoing research and stronger regulations to safeguard public health in the region.

Chemical waste dumping remains a significant environmental challenge in Africa. The Vaal River in South Africa, for instance, is widely regarded as one of the most polluted waterways in the region, with industrial effluents playing a major role in its contamination [298,306]. Although the South African government has initiated an ambitious cleanup project, addressing the scale of contamination remains a challenge. Similarly, the Nairobi River in Kenya has experienced episodes of industrial chemical dumping—at one point turning crimson red—raising serious concerns over potential toxic leaks. Residents have reported recurring instances of unusual water discoloration and an oily film on the water, prompting ongoing complaints against the responsible industries [300,304,306].

Efforts to improve landfill and chemical waste management are gaining momentum in Africa. In collaboration with the UNEP [288] and GEF [296], many countries are strengthening their national capacities for the safe management and elimination of hazardous chemicals and wastes. For example, South Africa has developed a comprehensive waste management strategy, that includes controlled landfills and a robust recycling framework, along with EPR [299,304] systems that hold manufacturers accountable for the entire lifecycle of their products. In Kenya, initiatives to enhance waste collection, promote the separation of recyclables, and adopt environmentally friendly disposal methods are being introduced, aiming to reduce landfill waste and support a circular economy [299,302].

Africa faces a multitude of environmental challenges, including deforestation, pollution, and climate change. Rapid urbanization and industrialization have increased the levels of pollution in air, water, and soil, while the region’s rich biodiversity is threatened by habitat destruction and resource overexploitation [300,302].

The enforcement of environmental regulations remains a significant hurdle. Despite the existence of policies, limited resources, corruption, and a lack of political will often hinder effective implementation and enforcement, resulting in ongoing environmental degradation. Moreover, the scarcity of comprehensive data and research further complicates efforts to understand and address the full scope of these environmental issues [300].

While the challenges are considerable, there is a growing commitment to sustainability across Africa. Increased collaboration among governments, NGOs, and international organizations is fostering innovative solutions to protect the environment. Investments in renewable energy, sustainable agriculture, and conservation initiatives are expected to drive future progress. Furthermore, advancements in bioremediation and pollution control technologies will be crucial for mitigating the impact of environmental contaminants, including PFASs [300,302].

## 5. Discussion

In analyzing “The PFAS Crisis and Colossal Catastrophic Systems Failure” issue using the Domino Effect Model, Swiss Cheese Model, and Ishikawa Fishbone Diagram, we can identify how multiple failures and gaps in various systems have led to the widespread contamination by and health impacts associated with PFAS compounds [307,308,309].

Systems accident analysis involves examining accidents within the context of their entire system, rather than focusing solely on individual components or human error [310].

This approach considers the interactions between technical, human, organizational, and environmental factors [310].

Models like the Domino Effect, Swiss Cheese Theory, and Ishikawa Fishbone Root Cause Analysis are used to identify systemic issues and improve safety [307,311,312].

The Domino Effect Model, developed by Herbert W. Heinrich, represents an accident sequence as a causal chain of events, similar to a row of dominos that topple in a chain reaction. The fall of the first domino leads to the fall of the second, followed by the third, and so on. This model emphasizes that a single cause is never sufficient to explain why an incident or injury took place. Instead, it highlights the importance of addressing multiple factors to prevent accidents [307].

The Swiss Cheese Model, developed by James Reason, illustrates how failures typically result from a combination of factors rather than a single root cause. It likens human systems to multiple slices of Swiss cheese, each with its holes representing weaknesses. When these holes align, a hazard passes through all layers, leading to failure. This model emphasizes the importance of having multiple layers of defense to prevent accidents [311].

Fishbone Root Cause Analysis, also known as the Ishikawa Diagram, is a visual tool used to identify the root causes of a problem. The main problem is placed at the “head” of the fish, and potential causes are categorized into branches, such as Methods, Machines, People, Materials, Measurements, and Environment. This method helps teams systematically explore and address the underlying issues [309].

Effective problem solving is an organized strategy to find, evaluate, and address challenges. Common techniques include the following:

1. Define the Problem: Clearly state the issue.
2. Brainstorm Solutions: Create a list of probable solutions.
3. Evaluate Solutions: Evaluate the feasibility and impact of each solution.
4. Implement the Solution: Put the selected solution into effect.
5. Monitor and Review: Evaluate the solution’s efficacy and make any necessary adjustments [313].

Combining systems accident analysis, Fishbone Root Cause Analysis, the Domino Effect Model, and the Swiss Cheese Theory can significantly enhance an organization’s ability to detect and successfully resolve issues.

By understanding the systemic nature of accidents, identifying root causes, and applying structured problem-solving methods, organizations can improve their safety, efficiency, and overall performance.

### 5.1. Domino Effect Model of Accident Causation

**Figure 4:**
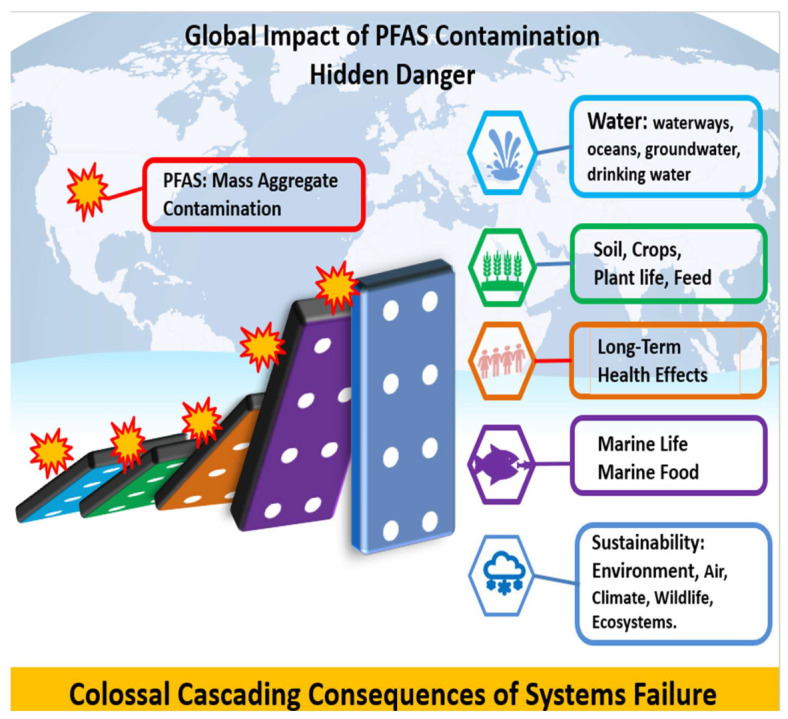
Domino Effect Model of accident causation.

The Domino Effect Model (Figure 4) suggests that a series of interconnected events or failures can lead to a larger catastrophic outcome. In the case of the PFAS crisis, the use of these chemicals in a wide range of products, combined with inadequate regulation and oversight, has created a chain reaction of contamination and health risks. The release of PFAS compounds into the environment has led to their accumulation in water sources, soil, and food supplies, resulting in widespread exposure and long-term health effects for humans and wildlife [314,315].

### 5.2. Swiss Cheese Model

**Figure 5:**
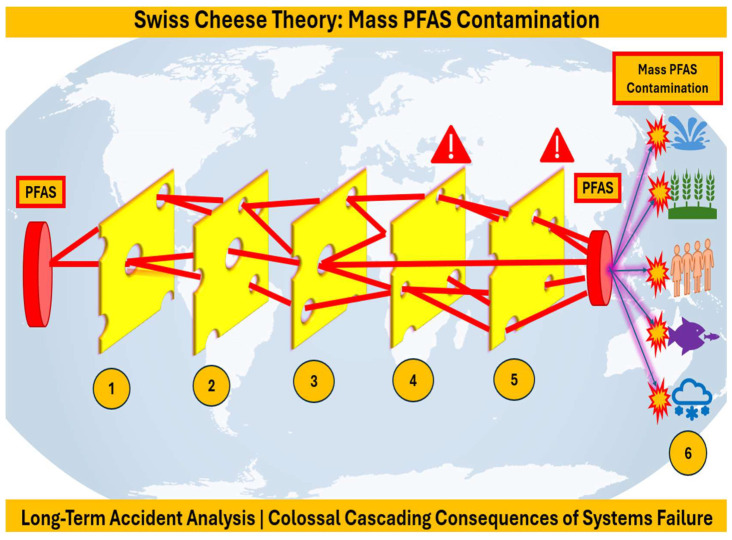
Long-term accident analysis—colossal cascading consequences of systems failure, Swiss Cheese Model.

**Figure 6:**
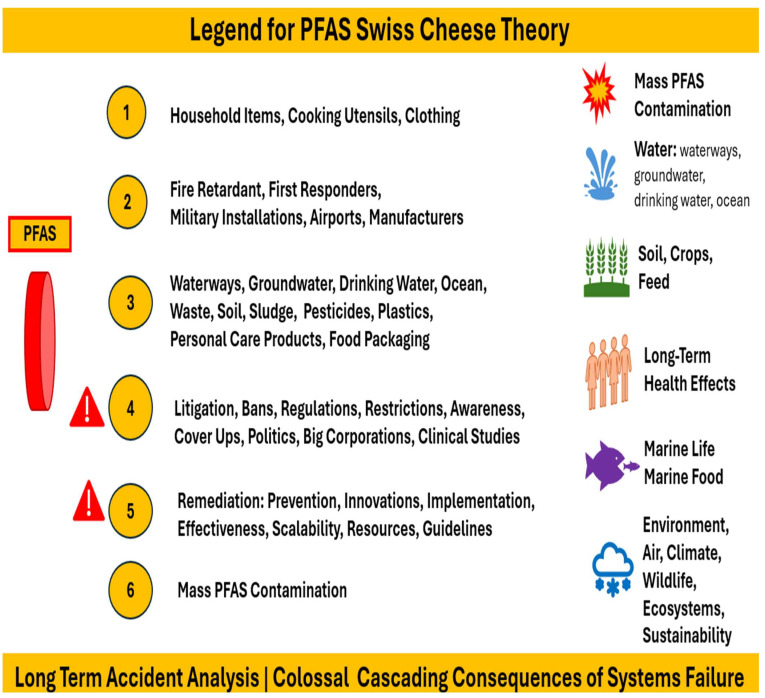
Legend for PFAS Swiss Cheese Theory systems failure.

The Swiss Cheese Model demonstrates how numerous layers of security or safeguards can have holes that, when aligned, allow an accident to occur. In the context of the PFAS crisis, regulatory failures, industry practices, and public awareness gaps have all contributed to the persistence of PFAS contamination. The lack of comprehensive regulations, ethical considerations by chemical companies, and potential cover-ups has allowed the problem to escalate and impact communities worldwide [308]. This is illustrated in Figure 5. The legend for PFAS Swiss Cheese Theory systems failure is outlined in Figure 6.

In addressing the PFAS crisis, potential bioremediation remedies, such as using microorganisms to break down PFAS compounds, offer a promising solution to mitigate contamination and reduce long-term health risks. However, further research and regulatory actions are needed to ensure the effectiveness and safety of these remediation methods.

By applying the Domino Effect Model and Swiss Cheese Model to the PFAS crisis, we can gain a deeper understanding of the complex interplay of factors that have contributed to this colossal system failure and work towards implementing comprehensive solutions to safeguard public health and the environment.

### 5.3. Ishikawa Fish Bone Root Cause Analyses

Ishikawa Fishbone Root Cause Analysis is a tool that visually maps probable contributing components to discover the root cause of an issue. In the context of the Global Forever Chemical PFAS Crisis, this method can be particularly useful for understanding the complex factors that have led to widespread PFAS contamination [316].

By systematically breaking down the problem and identifying contributing factors, Ishikawa Fishbone Root Cause Analysis helps stakeholders develop targeted and effective solutions to address the PFAS crisis [309].

The central issue or problem is the presence of PFASs (per- and polyfluoroalkyl substances) in the environment, particularly in water sources [317].

**Figure 7:**
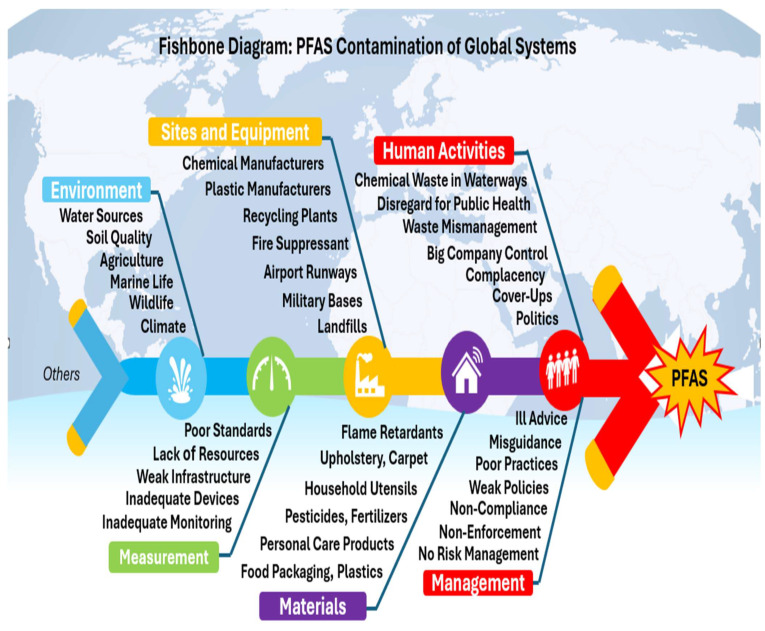
Fishbone diagram [311].

On the Fishbone Diagram, the horizontal line (the “spine”) represents the problem of PFAS contamination. Branching off this line are major categories of potential causes, “Human Activities”, “Management”, “Sites and Equipment”, “Materials”, “Environment”, and “Measurement”(Figure 7) [318].

For each category, brainstorming of possible causes of PFAS contamination has been carried out with regard to which materials and certain types of PFASs are used in existing products [319].

Sites and Equipment (Methods) refers to industrial processes that release PFASs [64] and equipment that may contribute to PFAS release [320].

Measurement refers to the inadequate monitoring of PFAS levels [317].

Human activities contributing to PFAS spread correspond to people [321].

Natural factors affecting PFAS distribution [292] have to do with the environment.

Policies and practices that may have allowed PFAS use [320] relate to their management. Waste Mismanagement refers to the improper disposal and management of PFAS-containing waste [267].

Hence, analysis and prioritization need to be implemented by evaluating the potential causes to determine which is most likely contributing to the problem. The prioritization of these causes will be achieved based on their impact and the feasibility of addressing them [318].

Based on this analysis, development strategies will be employed to mitigate or eliminate the root causes of PFAS contamination. Some suggested solutions are as follows:

The development and promotion of the use of safer alternative substances to replace PFASs in products [64].

The implementation of stricter regulations and best practices for industries to reduce PFAS emissions during manufacturing processes [317].

The upgrading of industrial equipment to prevent PFAS leakage and enhance the efficiency of PFAS capture technologies [322].

The establishment of comprehensive monitoring programs to regularly assess PFAS levels in the environment and identify contamination hotspots [321].

Of course, we need to increase public awareness and education on the sources and impacts of PFASs, encouraging responsible consumer behavior [319].

The implementation of remediation and cleanup efforts in contaminated areas, using advanced techniques like adsorption and filtration [64] needs to be considered.

Policies need to be strengthened, along with international agreements, to phase out the manufacturing and use of PFASs internationally [320]. Finally, waste management practices need to be improved to ensure the proper disposal and treatment of PFAS-containing waste, reducing environmental contamination [297].

Bioremediation, a cost-effective and eco-friendly method, can significantly contribute to meeting the Sustainable Development Goals (SDGs) by 2030. The cost of bioremediation varies, but it is generally lower than traditional remediation methods, with estimates ranging from USD 50.7 to USD 310.4 per m^3^ of contaminated soil [323]. Implementing bioremediation can help achieve the SDGs related to clean water and sanitation (Goal 6), sustainable cities and communities (Goal 11), and life below water (Goal 14) by reducing pollution and promoting environmental sustainability [324,325].

### 5.4. The Impact of PFASs on the Sustainable Development Goals (SDGs) of 2030

Addressing the PFAS crisis aligns with several United Nations Sustainable Development Goals (SDGs), including goals 3, 6, 12, 14, and 15.

Regarding Goal 3 and good health and well-being, reducing exposure to harmful chemicals improves public health and should be taken into account. The Centers for Disease Control and Prevention (CDC) and the Agency for Toxic Substances and Disease Registry (ATSDR) have conducted exposure assessments to determine the impact of PFASs on health. [14].

With regard to Goal 6 and clean water and sanitation, we need to ensure safe drinking water by mitigating PFAS contamination. The US Environmental Protection Agency (EPA) has set the first national drinking water guidelines for PFASs to safeguard communities from exposure [96].

Goal 12 refers to responsible consumption and production, hence encouraging sustainable practices and behaviors, and reducing chemical pollution (United Nations, 2015). The EPA has been investing in projects to address PFAS contamination in water through the Bipartisan Infrastructure Law [155].

With reference to Goal 14 and life below water, we need to protect marine life and ecosystems from PFAS contamination. The Waterkeeper Alliance has been working to monitor and remediate PFAS pollution in global waterways [325].

Finally, Goal 15 discusses life on land. Reducing PFAS contamination in soil and terrestrial ecosystems to protect biodiversity is vital. The OECD has hosted forums to address the environmental impact of PFASs [247].

By tackling the PFAS crisis through comprehensive regulation, public awareness, and individual actions, we can mitigate its impact and move towards a healthier, more sustainable future.

PFAS contamination is a global concern, affecting water supplies, soil, and air. These substances have been found in everything from groundwater and drinking water to the food chain, posing significant threats to food security and nutrition. The persistence of PFASs in the environment complicates remediation efforts, as these compounds do not degrade quickly and can spread across huge areas [326].

Researchers are exploring various bioremediation techniques to address PFAS contamination. These include using microbes and plants to absorb and break down PFAS compounds. Phytoremediation, for example, employs plants like birch and willow trees, which have shown potential in absorbing PFASs from the soil. Innovations in this area are critical for reducing PFAS levels at contaminated sites [327].

The future of PFAS management requires a multifaceted approach. This includes stricter regulations, investment in safer chemical alternatives, continuous monitoring and research, and public awareness about PFASs in consumer products. Sustainable manufacturing and waste management practices are necessary to mitigate these chemicals’ environmental footprint [317,328].

PFASs continue to be a substantial public health problem, prompting ongoing research and regulatory efforts. The CDC plays a vital function in studying the health effects of PFASs, guiding mitigation and prevention strategies, and supporting regulatory frameworks to protect communities from these persistent pollutants [92]. Continued monitoring and community engagement are vital to effective PFAS management [94,95,96].

## 6. PFASs, Medical Devices, and Other Industries

Evidence suggests the existence of PFASs in various consumer products and medical devices [329]. The exposure routes of PFASs in the general population could be cosmetics, food packaging, and personal hygiene products [330,331,332]. Organic fluorine was detected in most of the popular soft contact lens (CL) products, with concentrations ranging from 105 to 20,700 ppm [333,334], and the latter authors reported more pronounced differences in PFAS concentrations between CL users and non-users in females than in males.

They are also found in textiles, electronics, electric car batteries, pharmaceuticals, pesticides, and medical device manufacturing. Serious economic, industrial, environmental consequences, and, paradoxically, public health implications could arise from a blanket ban on all substances. Moreover, the replacement of PFASs with alternative substances may be expensive and even impossible [335].

Exposure to PFASs during waxing occurs for professional ski waxers [336]. A great risk of PFAS exposure with Aqueous Film-Forming Foam (AFFF) usage has been shown for firefighters. AFFFs have been used by firefighters since the 1960s in the extinguishment of chemical solvent- and hydrocarbon-fueled fires [337].

## 7. Conclusions

The PFAS crisis is a multifaceted issue, exacerbated by the mismanagement of waste, particularly in the context of wastewater treatment, landfills, and plastic production. The presence of PFAS chemicals in wastewater and landfills has caused their release into the environment, damaging water supplies and soil. Additionally, the use of PFASs in plastics has further contributed to the spread of these chemicals in the environment, impacting marine life and aquatic ecosystems.

Studies have shown that wastewater treatment plants are a significant source of PFAS contamination in water bodies. The persistence of these chemicals in wastewater effluents can lead to their accumulation in rivers, lakes, and oceans, posing risks and hazards to aquatic life and human health. Landfills also play an impactful role in the release of PFAS compounds into the environment, as leachate from landfills can contain high levels of these chemicals, contaminating groundwater and soil.

The use of PFASs in the production of plastics, such as food packaging and consumer goods, has led to the widespread distribution of these chemicals in the environment. When these plastic products are discarded or incinerated, PFAS compounds can be released into the air, water, and soil, contributing to pollution and environmental contamination.

The presence of PFAS compounds in water bodies has been shown to have detrimental effects on marine life and aquatic ecosystems. The bioaccumulation of PFASs in fish and other aquatic organisms can lead to health risks for both wildlife and humans who consume contaminated seafood. Additionally, the disruption of aquatic ecosystems due to PFAS contamination can have long-lasting impacts on biodiversity and ecosystem health.

In conclusion, the mismanagement of waste, including wastewater, landfills, and plastics, has significantly contributed to the PFAS crisis, leading to widespread contamination and environmental harm. Addressing these issues requires comprehensive regulatory actions, improved waste management practices, and sustainable alternatives to PFAS-containing products. Collaborative efforts involving government agencies, scientific research institutions, and environmental organizations are essential to mitigate the impacts of PFAS contamination and protect ecosystems and public health.

At the levels that are now ubiquitous in some environments, PFASs have a deleterious influence on human health. When there is a demonstrated benefit to human health and available, affordable, and effective technology, it makes sense to eliminate PFASs from the environment. A recent cost–benefit study supports the necessity for remediation, particularly for drinking water sources with high concentrations of PFASs, when the benefits to human health outweigh any potential drawbacks.

The future outlook for addressing PFAS contamination is centered on sustainability and comprehensive remediation strategies. Research has shown that PFAS compounds have significant health risks, necessitating a proactive approach to minimizing exposure and contamination.

Efforts to tackle the PFAS crisis have been bolstered by strategic initiatives, such as the US Environmental Protection Agency’s PFAS Strategic Roadmap, which contains commitments of action from 2021 to 2024. This roadmap emphasizes the importance of reducing PFAS emissions, enhancing detection methods, and accelerating the cleanup of contaminated sites.

One promising area of research is the bioremediation potential of plants to mitigate PFAS contamination. Studies have highlighted the ability of certain plant species to absorb and break down PFAS compounds, offering a cost-effective and sustainable solution for environmental remediation.

The C8 Science Panel’s assessment of the likely relationships between PFOA exposure and human health effects further underscores the need for stringent regulatory measures and ongoing monitoring to protect public health.

“The ever-increasing mass of PFASs in the global environment necessitates a change in the mass balance, either through increased remediation, reduced emissions, or both”. This review explores the potential costs (to human health, ecosystems, climate, future generations, sustainability, food security, etc.) of relying entirely on increased remediation without decreasing PFAS consumption and emissions. The anticipated annual costs of removing PFASs from the environment at the current rate of emission range from USD 20 to 7000 trillion. Without major reductions in production and emissions, the costs are anticipated to exceed the world GDP of USD 106 trillion, making it unfeasible to manage PFAS pollution through remediation alone.

The only method available to address the mass of PFASs steadily accumulating in the environment is to alter the mass balance by reducing emissions, increasing removal, or accomplishing both. Integrating sustainable practices within food systems is crucial for supporting a healthy planet. Sustainable food systems can help mitigate PFAS contamination by promoting eco-friendly agricultural practices and reducing the use of harmful chemicals. This approach not only addresses current environmental issues but also supports long-term food security and public health. This review adds to the ongoing discourse on PFAS policy by focusing more heavily on remediation.

## Figures and Tables

**Table 1:** Center for Disease Control (CDC) PFAS Medical Studies and Guidelines [93,94,95,96,97].

Recommendations Key Findings:	In Response to the Health Hazards Linked with PFASs, the CDC Provides Various Guidelines:
**Health Effects**	**Preventive Measures**
Research reveals an association between PFAS exposure and a variety of health concerns, including immune system effects, hormonal disruption, and increased cholesterol levels. Studies have also suggested possible links to certain cancers, such as kidney and testicular cancer.	The CDC advises limiting the use of products containing PFASs, especially for everyday items like water-resistant fabrics and non-stick cookware. Consumer education on identifying and avoiding PFAS products is critical
**Biomonitoring**	**Remediation Strategies**
The CDC’s National Health and Nutrition Examination Survey (NHANES) has studied PFAS levels in the human population, providing essential data on exposure levels across the US population.	For contaminated sites, the CDC recommends various remediation strategies, including the following: Activated Carbon Filtration: Effective in reducing PFAS concentrations in drinking water. Ion Exchange Resins: Used to absorb PFAS from water supplies. High-Temperature Incineration: Identified as a method for breaking down PFASs in waste materials.
**Community Studies**	
The CDC’s Agency for Toxic Substances and Disease Registry (ATSDR) has undertaken health assessments in communities with potential PFAS pollution, allowing researchers to better understand the localized health effects.	
**Policy Recommendations**	
The CDC calls for stricter regulations governing PFAS use and disposal, emphasizing the need for states and local entities to create water quality standards that reflect the most recent scientific findings regarding PFAS toxicity (CDC Policy Recommendations for PFAS Regulation, 2020).	

**Table 2:** PFASs in drinking water and food [96,154,155,156,157,158,159,160,161,162,163,164,165,166].

Supplies	Contaminant Sources	Risks, Exposure	Mitigation	Areas of Concerns	Regulatory Needs
**Water:** **Drinking/** **Potable**	Chemical Waste Landfill leaching Farmland sludge Plastic islands	Public health Soil health Agri-food systems Cattle and poultry Food processing	Rapid tests: Rapid PFAS test kits would be ideal for the food industry and home use, improving monitoring capabilities at critical points.	The lack of funding and resources for the widespread implementation of PFAS testing, mitigation, and bioremediation in the food industry and water processing and treatment sites.	EPA Final PFAS National Primary Drinking Water Regulation, April 2024 https://www.epa.gov/sdwa/and-polyfluoroalkyl-substances-pfas (accessed on 25 February 2025)
**Food Products**	Food contact materials	Health effect risks: ‣ Endocrine disruptors, ‣ carcinogens, ‣ gastrointestinal disorders ‣ neurological disorders Developmental disorders: Due to children’s developing bodies, they are especially susceptible to chemical contaminants.	Safer packaging alternatives:	A primary concern is the persistent utilization of detrimental chemicals in food packaging. Bisphenol-A (BPA) and PFASs continue to be extensively utilized despite their recognized hazards. Studies indicate that alternative materials, including glass and polyethylene terephthalate (PET), can reduce the transfer of hazardous chemicals into food. Nevertheless, the extensive implementation of these materials will necessitate substantial alterations in manufacturing methodologies and consumer habits.	Stricter regulations: Global regulatory agencies have enacted numerous regulations to restrict chemical contamination in food. The EFSA has established tolerable daily intake (TDI) thresholds for numerous hazardous substances. However, these policies vary significantly between areas, with developing countries generally having less comprehensive regulatory frameworks. This inequality in enforcement puts some populations at risk of being exposed to unsafe quantities of pollutants.

## Data Availability

No new data were created or analyzed in this study.

## Supplementary Materials

The following supporting information can be downloaded at: https://www.mdpi.com/article/10.3390/foods14060958/s1, Table S1: PFAS Concentrations in Food and Dietary intake in several countries [99,100,102,111,113,119,121,122,139,140,141,142,143,144,145,146,147,148,149,150,151,152].

## Glossary of Acronyms

ABM	Agent-Based Model;
ASEAN	Association of Southeast Asian Nations;
ATSDR	Agency for Toxic Substances and Disease Registry;
AU	African Union;
BAFs	Bioaccumulation efficiencies;
BPA	Bisphenol-A;
CDC	Center for Disease Control;
CEPA	Canadian Environmental Protection Act;
CFCIP	Changshu fluorine-chemical industrial park;
CIHR	Canadian Institutes of Health Research;
ECCC	Environment and Climate Change Canada;
ECHA	European Chemicals Agency;
EDCs	Endocrine-disrupting chemicals;
EFSA	European Food Safety Authority;
EPA	Environmental Protection Agency;
EPR	Extended Producer Responsibility;
EQS	Environmental Quality Standard;
FSANZ	Food Standards Australia New Zealand;
GEF	Global Environment Facility;
HFPO-DA	Hexafluoropropylene oxide-dimer acid;
HPLC-MS/MS	High-performance liquid chromatography–tandem mass spectrometry;
HR	Hazard ratio;
HRMS	High-resolution mass spectrometry;
IDB	Inter-American Development Bank;
IPEN	International Pollutants Elimination Network;
iPSC	Induced pluripotent stem cell;
LC-HRMS	Liquid chromatography coupled with high-resolution mass spectrometry;
LC-Q-Orbitrap	LC coupled to a hybrid high-resolution mass analyzer;
LC–MS/MS	Liquid chromatography–tandem mass spectrometry;
MALDI-MS	Matrix-assisted laser desorption/ionization mass spectrometry;
Mercosur	Mercado Común del Sur;
MOIT	Ministry of Industry and Trade;
MONRE	Ministry of Natural Resources and Environment;
NGO	Non-governmental organization;
NHANES	National Health and Nutrition Examination Survey;
NCP	Northern Contaminants Program;
NRCan	Natural Resources Canada;
OECD	Organization for Economic Co-operation and Development;
PBK	Physiologically Based Kinetic;
PFDoDA	Perfluoro-n-dodecanoic acid;
PFPeA	Perfluorinated acid;
PFAS	Polyfluoroalkyl substance;
PFBA	Alternative perfluorobutanoic acid;
PFBS	Perfluorobutane sulfonate;
PFCA	Perfluoroalkyl carboxylic acid;
PFHxS	Perfluorohexane sulfonate;
PFHpA	Perfluoroheptanoic acid;
PFHxA	Perfluorohexanoic acid;
PFNA	Perfluorononanoic acid;
PFOA	Perfluorooctanoic acid;
PFOS	Perfluorooctane sulfonic acid;
PET	Polyethylene terephthalate;
PFTrDA	Perfluorotridecanoic acid;
PFUnDA	Perfluoropentanoic acid;
PMRA	Pest Management Regulatory Agency;
POP	Persistent organic pollutant;
RAPIMER	Renewable Artificial Plant for In Situ Microbial Environmental Remediation;
TDI	Tolerable daily intake;
TDS	Total Diet Study;
UHPLC-MS/MS	Ultra-high performance liquid chromatography–tandem mass spectrometry;
UNEP	United Nations Environment Programme;
UPLC-TQS	Ultraperformance liquid chromatography–triple-quadrupole mass spectrometry;
WSER	Wastewater systems effluent regulation;
WWTP	Wastewater treatment plant.

## References

[1] Interstate Technology Regulatory Council (ITRC) (2020). PFAS History and Use. https://pfas-1.itrcweb.org/wp-content/uploads/2020/10/history_and_use_508_2020Aug_Final.pdf.

[2] Environmental Working Group (EWG) (2021). Timeline: The PFOA Saga. https://www.ewg.org/research/timeline-forever-chemicals-and-firefighters.

[3] Union of Concerned Scientists UCSUSA (2019). Dark Waters—The Lawyer Who Became DuPont’s Worst Nightmare. https://blog.ucsusa.org/genna-reed/dark-waters-speaks-the-truth-about-pfas/.

[4] NIH US National Library of Medicine (2021). PFAS and Health Effects. https://www.ncbi.nlm.nih.gov/books/NBK584690/.

[5] Lerner S. (2024). How 3M Executives Convinced a Scientist the Forever Chemicals She Found in Human Blood Were Safe. https://www.propublica.org/article/3m-forever-chemicals-pfas-pfos-inside-story.

[6] Pruitt S. (2020). The Post World War II Boom: How America Got into Gear. https://www.history.com/news/post-world-war-ii-boom-economy.

[7] Agency for Toxic Substances and Disease Registry ATSDR (2022). PFAS Exposure Assessments. https://www.atsdr.cdc.gov/pfas/exposure-assessments/index.html.

[8] Interstate Technology Regulatory Council ITRC (2023). 9 Site Risk Assessment—PFAS—Per- and Polyfluoroalkyl Substances. https://pfas-1.itrcweb.org/9-site-risk-assessment/.

[9] Balbuena N., DiFelice M. (2023). How Chemical Makers Hid the Truth About PFAS. https://www.foodandwaterwatch.org/2023/11/08/pfas-coverups/.

[10] Tullo A. (2021). C&EN’s Top 50 US Chemical Producers for 2021. https://cen.acs.org/business/finance/CENs-top-50-US-chemical-producers-for-2020/99/i17.

[11] Reed G. (2019). DuPont’s Worst Nightmare. https://www.theguardian.com/commentisfree/2019/dec/27/chemicals-dupont-rob-bilott-toxic-america.

[12] Smith J., Doe A., White R. (2020). Interactions between PFAS and Microplastics in the Environment. Environ. Sci. Technol..

[13] Jones L., Brown M. (2021). The PFAS and Microplastics Nexus: Implications for Public Health and Environmental Policy. J. Environ. Manag..

[14] Agency for Toxic Substances and Disease Registry Per- and Polyfluoroalkyl Substances (PFAS) and Your Health. 18 January 2024. https://www.atsdr.cdc.gov/pfas/index.html.

[15] National Oceanic and Atmospheric Administration Microplastics and the Environment: Research and Guidelines|National Oceanic and Atmospheric Administration. 10 June 2024. https://marinedebris.noaa.gov/search-md-website?search_api_fulltext=microplastics+and+the+environment+research+and+guidelines.

[16] Bakir A., Rowland S.J., Thompson R.C. (2012). Competitive sorption of persistent organic pollutants onto microplastics in the marine environment. Mar. Pollut. Bull..

[17] Wang X., Wang Y., Li J., Liu J., Zhao Y., Wu Y. (2021). Occurrence and dietary intake of Perfluoroalkyl substances in foods of the residents in Beijing, China. Food Addit. Contam. Part B.

[18] Rochman C.M., Tahir A., Williams S.L., Baxa D.V., Lam R., Miller J.T., Teh F.C., Werorilangi S., Teh S.J. (2015). Anthropogenic debris in seafood: Plastic debris and fibers from textiles in fish and bivalves sold for human consumption. Sci. Rep..

[19] Fossi M.C., Marsili L., Baini M., Giannetti M., Coppola D., Guerranti C., Caliani I., Minutoli R., Lauriano G., Finoia M.G. (2016). Fin whales and microplastics: The Mediterranean Sea and the Sea of Cortez scenarios. Environ. Pollut..

[20] Toms L.M.L., Calafat A.M., Kato K., Thompson J., Harden F., Hobson P., Sjodin A., Mueller J.F. (2009). Polyfluoroalkyl chemicals in pooled human serum from Australia in 2002, 2004, 2006, and 2008. Environ. Sci. Technol..

[21] Teuten E.L., Saquing J.M., Knappe D.R., Barlaz M.A., Jonsson S., Björn A., Rowland S.J., Thompson R.C., Galloway T.S., Yamashita R. (2009). Transport and release of chemicals from plastics to the environment and to wildlife. Philosophical Transactions of the Royal Society. Biol. Sci..

[22] National Oceanic and Atmospheric Administration NOAA (2024). Addressing the Challenges of Plastics and PFAS. https://marinedebris.noaa.gov/research/influence-environmental-conditions-contaminants-leaching-and-sorbing-marine-microplastic.

[23] Merkl A., Charles D. (2022). The Price of Plastic Pollution: Social Costs and Corporate Liabilities. https://www.beyondplastics.org/reports/the-price-of-plastic-pollution.

[24] Minnesota Pollution Control Agency Groundbreaking Study Shows Unaffordable Costs of PFAS Cleanup from Wastewater. 6 June 2023. https://www.pca.state.mn.us/news-and-stories/groundbreaking-study-shows-unaffordable-costs-of-pfas-cleanup-from-wastewater.

[25] EPA US Environmental Protection Agency (2024). PFAS Explained|US EPA. https://www.epa.gov/pfas/pfas-explained.

[26] Ao J., Yuan T., Xia H., Ma Y., Shen Z., Shi R. (2019). Characteristic and human exposure risk assessment of per- and polyfluoroalkyl substances: A study based on indoor dust and drinking water in China. Environ. Pollut..

[27] Cao H., Zhou Z., Hu Z., Wei C., Li J., Wang L. (2022). Effect of Enterohepatic Circulation on the Accumulation of Per- and Polyfluoroalkyl Substances: Evidence from Experimental and Computational Studies. Environ. Sci Technol..

[28] Jane L., Espartero L., Yamada M., Ford J., Owens G., Prow T., Juhasz A. (2022). Health-related toxicity of emerging per- and polyfluoroalkyl substances: Comparison to legacy PFOS and PFOA. Environ. Res..

[29] Amstutz V.H., Cengo A., Gehres F., Sijm D.T.H.M., Vrolijk M.F. (2022). Investigating the cytotoxicity of per- and polyfluoroalkyl substances in HepG2 cells: A structure-activity relationship approach. Toxicology.

[30] Ojo A.F., Xia Q., Peng C., Ng J.C. (2021). Evaluation of the individual and combined toxicity of perfluoroalkyl substances to human liver cells using biomarkers of oxidative stress. Chemosphere.

[31] Solan M.E., Senthilkumar S., Aquino G.V., Bruce E.D., Lavado R. (2022). Comparative cytotoxicity of seven per- and polyfluoroalkyl substances (PFAS) in six human cell lines. Toxicology.

[32] Xu M., Wan J., Niu Q., Liu R. (2019). PFOA and PFOS interact with superoxide dismutase and induce cytotoxicity in mouse primary hepatocytes: A combined cellular and molecular methods. Environ. Res..

[33] Xu M., Liu G., Li M., Huo M., Zong W., Liu R. (2020). Probing the cell apoptosis pathway induced by perfluorooctanoic acid and perfluorooctane sulfonate at the subcellular and molecular levels. J. Agric. Food Chem..

[34] Donat-Vargas C., Bergdahl I.A., Tornevi A., Wennberg M., Sommar J., Koponen J. (2019). Associations between repeated measure of plasma perfluoroalkyl substances and cardiometabolic risk factors. Environ. Int..

[35] Huang M., Jiao J., Zhuang P., Chen X., Wang J., Zhang Y. (2018). Serum polyfluoroalkyl chemicals are associated with risk of cardiovascular diseases in national US population. Environ. Int..

[36] Conley J.M., Lambright C.S., Evans N., McCord J., Strynar M.J., Hill D. (2021). Hexafluoropropylene oxide-dimer acid (HFPO-DA or GenX) alters maternal and fetal glucose and lipid metabolism and produces neonatal mortality, low birthweight, and hepatomegaly in the Sprague-Dawley rat. Environ. Int..

[37] Moro G., Liberi S., Vascon F., Linciano S., De Felice S., Fasolato S. (2022). Investigation of the interaction between human serum albumin and branched short-chain perfluoroalkyl compounds. Chem. Res. Toxicol..

[38] Solan M.E., Koperski C.P., Senthilkumar S., Lavado R. (2023). Short-chain per- and polyfluoralkyl substances (PFAS) effects on oxidative stress biomarkers in human liver, kidney, muscle, and microglia cell lines. Environ. Res..

[39] Wen Y., Mirji N., Irudayaraj J. (2020). Epigenetic toxicity of PFOA and GenX in HepG2 cells and their role in lipid metabolism. Toxicol. In Vitro.

[40] Yang Y.D., Li J.X., Lu N., Tian R. (2023). Serum albumin mitigated perfluorooctane sulfonate-induced cytotoxicity by affecting the cellular responses. Biophys. Chem..

[41] Yang Y.D., Tian R., Lu N. (2023). Binding of serum albumin to perfluorooctanoic acid reduced cytotoxicity. Sci. Total Environ..

[42] Alesio J.L., Slitt A., Bothun G.D. (2022). Critical new insights into the binding of poly- and perfluoroalkyl substances (PFAS) to albumin protein. Chemosphere.

[43] Bangma J., Szilagyi J., Blake B.E., Plazas C., Kepper S., Fenton S.E. (2020). An assessment of serum-dependent impacts on intracellular accumulation and genomic response of per- and polyfluoroalkyl substances in a placental trophoblast model. Environ. Toxicol..

[44] Beesoon S., Martin J.W. (2015). Isomer-specific binding affinity of perfluorooctanesulfonate (PFOS) and perfluorooctanoate (PFOA) to serum proteins. Environ. Sci. Technol..

[45] Forsthuber M., Kaiser A.M., Granitzer S., Hassl I., Hengstschläger M., Stangl H., Gundacker C. (2020). Albumin is the major carrier protein for PFOS, PFOA, PFHxS, PFNA and PFDA in human plasma. Environ. Int..

[46] Chi Q., Li Z., Huang J., Ma J., Wang X. (2018). Interactions of perfluorooctanoic acid and perfluorooctanesulfonic acid with serum albumins by native mass spectrometry, fluorescence and molecular docking. Chemosphere.

[47] Crisalli A.M., Cai A., Cho B.P. (2023). Probing the Interactions of Perfluorocarboxylic Acids of Various Chain Lengths with Human Serum Albumin: Calorimetric and Spectroscopic Investigations. Chem. Res. Toxicol..

[48] Jackson T.W., Scheibly C.M., Polera M.E., Belcher S.M. (2021). Rapid characterization of human serum albumin binding for per- and polyfluoroalkyl substances using differential scanning fluorimetry. Environ. Sci. Technol..

[49] Qin P., Liu R., Pan X., Fang X., Mou Y. (2010). Impact of carbon chain length on binding of perfluoroalkyl acids to bovine serum albumin determined by spectroscopic methods. J. Agric. Food Chem..

[50] Zhang R., Zhang H., Chen B., Luan T. (2020). Fetal bovine serum attenuating perfluorooctanoic acid-inducing toxicity to multiple human cell lines via albumin binding. J. Hazard. Mater..

[51] Peng S.-Y., Yang Y.-D., Tian R., Lu N. (2025). Critical new insights into the interactions of hexafluoropropylene oxide-dimer acid (GenX or HFPO-DA) with albumin at molecular and cellular levels. J. Environ. Sci..

[52] Rafiee A., Faridi S., Sly P.D., Stone L., Kennedy L.P., Mahabee-Gittens E.M. (2024). Asthma and decreased lung function in children exposed to perfluoroalkyl and polyfluoroalkyl substances (PFAS): An updated meta-analysis unveiling research gaps. Environ. Res..

[53] Starnes H.M., Rock K.D., Jackson T.W., Belcher S.M. (2022). A critical review and meta- analysis of impacts of per- and polyfluorinated substances on the brain and behavior. Front. Toxicol.

[54] Haug L.S., Huber S., Becher G., Thomsen C. (2011). Characterisation of human exposure pathways to perfluorinated compounds—Comparing exposure estimates with biomarkers of exposure. Environ. Int..

[55] Daly E.R., Chan B.P., Talbot E.A., Nassif J., Bean C., Cavallo S.J., Metcalf E., Simone K., Woolf A.D. (2018). Per- and polyfluoroalkyl substance (PFAS) exposure assessment in a community exposed to contaminated drinking water, New Hampshire, 2015. Int. J. Hyg. Environ. Health.

[56] Graber J.M., Alexander C., Laumbach R.J., Black K., Strickland P.O., Georgopoulos P.G., Marshall E.G., Shendell D.G., Alderson D., Mi Z. (2019). Per and polyfluoroalkyl substances (PFAS) blood levels after contamination of a community water supply and comparison with 2013–2014 NHANES. J. Expo. Sci. Environ. Epidemiol..

[57] Rocabois A., Sanchez M., Philippat C., Crépet A., Wies B., Vrijheid M., Nieuwenhuijsen M., Slama R. (2024). Chemical exposome and children health: Identification of dose-response relationships from meta-analyses and epidemiological studies. Environ. Res..

[58] Schillemans T., Donat-Vargas C., Akesson A. (2024). Per- and polyfluoroalkyl sub¬stances and cardiometabolic diseases: A review. Basic Clin. Pharmacol. Toxicol..

[59] Ford L.C., Lin H.-C., Zhou Y.-H., Wright F.A., Gombar V.K., Sedykh A., Shah R.R., Chiu W.A., Rusyn I. (2024). Characterizing PFAS hazards and risks: A human population-based in vitro cardiotoxicity assessment strategy. Hum. Genom..

[60] Cornelis C., D’Hollander W., Roosens L., Covaci A., Smolders R., Van Den Heuvel R., Govarts E., Van Campenhout K., Reynders H., Bervoets L. (2012). First assessment of population exposure to perfluorinated compounds in Flanders, Belgium. Chemosphere.

[61] Stubleski J., Salihovic S., Lind L., Lind P.M., van Bavel B., Kärrman A. (2016). Changes in serum levels of perfluoroalkyl substances during a 10-year follow-up period in a large population-based cohort. Environ. Int..

[62] Sifakis S., Androutsopoulos V.P., Tsatsakis A.M., Spandidos D.A. (2017). Human exposure to endocrine disrupting chemicals: Effects on the male and female reproductive systems. Environ. Toxicol. Pharmacol..

[63] Smarr M.M., Kannan K., Buck Louis G.M. (2016). Endocrine disrupting chemicals and endometriosis. Fertil. Steril..

[64] Cousins F.L., McKinnon B.D., Mortlock S., Fitzgerald H.C., Zhang C., Montgomery G.W., Gargett C.E. (2023). New concepts on the etiology of endometriosis. J. Obstet. Gynaecol. Res..

[65] de Haro-Romero T., Peinado F.M., Vela-Soria F., Lara-Ramos A., Fernández-Parra J., Molina-Lopez A., Ubiña A., Ocón O., Artacho-Cordón F., Freire C. (2024). Association between exposure to perfluoroalkyl substances (PFAS) and endometriosis in the ENDEA case-control study. Sci. Total Environ..

[66] Giesy J.P., Kannan K., Jones P.D., Hilscherova K. (2004). Perfluorinated chemicals in the environment: A review. Rev. Environ. Contam. Toxicol..

[67] Hammarstrand S., Jakobsson K., Andersson E., Xu Y., Li Y., Olovsson M., Andersson E.M. (2021). Perfluoroalkyl substances (PFAS) in drinking water and risk for polycystic ovarian syndrome, uterine leiomyoma, and endometriosis: A Swedish cohort study. Environ. Int..

[68] Li Y., Fletcher T., Mucs D., Scott K., Lindh C.H., Tallving P., Jakobsson K. (2018). Half-lives of PFOS, PFHxS and PFOA after end of exposure to contaminated drinking water. Occup. Environ. Med..

[69] Rickard B.P., Rizvi I., Fenton S.E. (2022). Per- and poly-fluoroalkyl substances (PFAS) and female reproductive outcomes: PFAS elimination, endocrine-mediated effects, and disease. Toxicology.

[70] Bjerve K., Småstuen L., Thomsen C., Sabaredzovic A., Becher G., Brunborg G. (2012). Placental transfer of perfluorinated compounds is selective—A Norwegian Mother and Child sub-cohort study. Int. J. Hyg. Environ. Health.

[71] Domínguez-Liste A., de Haro-Romero T., Quesada-Jim’enez R., P’erez-Cantero A., Peinado F.M., Ballesteros Ó., Vela-Soria F. (2024). Multiclass determination of endocrine-disrupting chemicals in meconium: First evidence of Perfluoroalkyl substances in this biological compartment. Toxics.

[72] Kim Y.R., White N., Bräunig J., Vijayasarathy S., Mueller J.F., Knox C.L., Harden F.A., Pacella R., Toms L.M.L. (2020). Per- and poly-fluoroalkyl substances (PFASs) in follicular fluid from women experiencing infertility in Australia. Environ. Res..

[73] Vela-Soria F., Serrano-L’opez L., García-Villanova J., de Haro T., Olea N., Freire C. (2020). HPLC-MS/MS method for the determination of perfluoroalkyl substances in breast milk by combining salt-assisted and dispersive liquid-liquid microextraction. Anal. Bioanal. Chem..

[74] Zheng P., Liu Y., An Q., Yang X., Yin S., Ma L.Q., Liu W. (2022). Prenatal and postnatal exposure to emerging and legacy per-/poly fluoroalkyl substances: Levels and transfer in maternal serum, cord serum, and breast milk. Sci. Total Environ..

[75] Fabelova L., Beneito A., Casas M., Colles A., Dalsager L., Den Hond E., Dereumeaux C., Ferguson K., Gilles L., Govarts E. (2023). PFAS levels and exposure determinants in sensitive population groups. Chemosphere.

[76] Freire C., Vela-Soria F., Castiello F., Salamanca-Fernández E., Quesada-Jiménez R., López-Alados M.C., Fernandez M.F., Olea N. (2023). Exposure to perfluoroalkyl substances (PFAS) and association with thyroid hormones in adolescent males. Int. J. Hyg. Environ. Health.

[77] McAdam J., Bell E.M. (2023). Determinants of maternal and neonatal PFAS concentrations: A review. Environ. Health.

[78] Richterova D., Govartsb E., Fabelova L., Rausova K., Martin L.R., Gilles L., Remy S., Colles A., Rambaudc L., Riouc M. (2023). PFAS levels and determinants of variability in exposure in European teenagers—Results from the HBM4EU aligned studies (2014–2021). Int. J. Hyg. Environ. Health.

[79] Zhang Y., Mustieles V., Wang Y., Sun Y., Agudelo J., Bibi Z., Torres N., Oulhote Y., Slitt A., Messerlian C. (2023). Folate concentrations and serum perfluoroalkyl and polyfluoroalkyl substance concentrations in adolescents and adults in the USA (National Health and Nutrition Examination Study 2003–2016): An observational study. Lancet Planet. Health.

[80] Wu Y., Qiu Y., Wu Y., Li H., Yang H., Deng Q., He B., Yan F., Li Y., Chen F. (2024). Association of per- and polyfluoroalkyl substances (PFAS) with periodontitis: The mediating role of sex hormones. BMC Oral Health.

[81] Zhou X., Wang X., Ou T., Huang L., He B. (2024). Association between family economic situation and serum PFAS concentration in American adults with hypertension and hyperlipemia. Sci. Rep..

[82] Koulini G.V., Nambi I.M. (2024). Occurrence of forever chemicals in Chennai waters, India. Environ. Sci. Eur..

[83] Belmaker I., Anca E.D., Rubin L.P., Magen-Molho H., Miodovnik A., van der Hal N. (2024). Adverse health effects of exposure to plastic, microplastics and their additives: Environmental, legal and policy implications for Israel. Isr. J. Health Policy Res..

[84] Biggeri A., Stoppa G., Facciolo L., Fin G., Mancini S., Manno V., Minelli G., Zamagni F., Zamboni M., Catelan D. (2024). All-cause, cardiovascular disease and cancer mortality in the population of a large Italian area contaminated by perfluoroalkyl and polyfluoroalkyl substances (1980–2018). Environ. Health.

[85] Schrenk D., Bignami M., Bodin L., Chipman J.K., del Mazo J., Grasl-Kraupp B., Hogstrand C., Hoogenboom L., Leblanc J.C., EFSA Panel on Contaminants in the Food Chain (EFSA CONTAM Panel) (2020). Risk to human health related to the presence of perfluoroalkyl substances in food. EFSA J..

[86] Iulini M., Russo G., Crispino E., Paini A., Fragki S., Corsini E., Pappalardo F. (2024). Advancing PFAS risk assessment: Integrative approaches using agent-based modeling and physiologically based kinetic for environmental and health safety. Comput. Struct. Biotechnol. J..

[87] Pappalardo F., Russo G., Corsini E., Paini A., Worth A. (2022). Translatability and transferability of in silico models: Context of use switching to predict the effects of environmental chemicals on the immune system. Comput. Struct. Biotechnol. J..

[88] Ehrlich V., Bil W., Vandebriel R., Granum B., Luijten M., Lindeman B., Grandjean P., Kaiser A.M., Hauzenberger I., Hartmann C. (2023). Consideration of pathways for immunotoxicity of per-and polyfluoroalkyl substances (PFAS). Environ. Health.

[89] Perera D.C., Meegoda J.N. (2024). PFAS: The Journey from Wonder. Chemicals to Environmental Nightmares and the Search for Solutions. Appl. Sci..

[90] Zhang Y., Beesoon S., Zhu L., Martin J.W. (2013). Biomonitoring of perfluoroalkyl acids in human urine and estimates of biological half-life. Environ. Sci. Technol..

[91] Langenbach B., Wilson M. (2021). Per- and polyfluoroalkyl substances (PFAS): Significance and considerations within the regulatory framework of the USA. Int. J. Environ. Res. Public Health.

[92] Safta D. (2024). Per- and Polyfluorinated Substances (PFAS); a Literature Review. Undergrad. J. Public Health.

[93] ATSDR (2016). Community Health Assessments, 18 October|Agency for Toxic Substances and Disease Registry. https://www.atsdr.cdc.gov/hac/index.html.

[94] CDC (2024). PFAS Information for Clinicians. https://www.atsdr.cdc.gov/pfas/hcp/clinical-overview/human-exposure.html.

[95] EPA (2024). Key EPA Actions to Address PFAS 1 September. https://www.epa.gov/pfas/key-epa-actions-address-pfas.

[96] EPA (2024). Fact Sheet Treatment Options from Drinking Water. https://www.epa.gov/system/files/documents/2024-04/pfas-npdwr_fact-sheet_treatment_4.8.24.pdf.

[97] ATSDR, Agency for Toxic Substances and Disease Registry PFAS in the U.S. Population. https://www.atsdr.cdc.gov/pfas/data-research/facts-stats/index.html.

[98] Starling M.C.V., Rodrigues D.A., Miranda G.A., Jo S., Amorim C.C., Ankley G.T., Simcik M. (2024). Occurrence and potential ecological risks of PFAS in Pampulha Lake, Brazil, a UNESCO world heritage site. Sci. Total Environ..

[99] Wang Y., Gao X., Liu J., Lyu B., Li J., Zhao Y., Wu Y. (2022). Exposure to Emerging and Legacy Polyfluoroalkyl Substances in the Sixth Total Diet Study—China, 2016–2019. China CDC Wkly..

[100] Bao J., Yu W.J., Liu Y., Wang X., Jin Y.H., Dong G.H. (2019). Perfluoroalkyl substances in groundwater and home-produced vegetables and eggs around a fluorochemical industrial park in China. Ecotoxicol. Environ. Saf..

[101] Scher D.P., Kelly J.E., Huset C.A., Barry K.M., Hoffbeck R.W., Yingling V.L., Messing R.B. (2018). Occurrence of perfluoroalkyl substances (PFAS) in garden produce at homes with a history of PFAS-contaminated drinking water. Chemosphere.

[102] Bao J., Li C.-L., Liu Y., Wang X., Yu W.-J., Liu Z.-Q., Shao L.-X., Jin Y.-H. (2020). Bioaccumulation of perfluoroalkyl substances in greenhouse vegetables with long-term groundwater irrigation near fluorochemical plants in Fuxin, China. Environ. Res..

[103] Bao J., Liu W., Liu L., Jin Y., Dai J., Ran X., Zhang Z., Tsuda S. (2011). Perfluorinated compounds in the environment and the blood of residents living near fluorochemical plants in Fuxin, China. Environ. Sci. Technol..

[104] Xu C., Liu Z., Song X., Ding X., Ding D. (2021). Legacy and emerging per-and polyfluoroalkyl substances (PFASs) in multi-media around a landfill in China: Implications for the usage of PFASs alternatives. Sci. Total Environ..

[105] Zhou Y., Wang T., Jiang Z., Kong X., Li Q., Sun Y., Wang P., Liu Z. (2017). Ecological effect and risk towards aquatic plants induced by perfluoroalkyl substances: Bridging natural to culturing flora. Chemosphere.

[106] Tan K.-Y., Lu G.-H., Yuan X., Zheng Y., Shao P.-W., Cai J.-Y., Zhao Y.-R., Zhu X.-H., Yang Y.-L. (2018). Perfluoroalkyl substances in water from the Yangtze River and its tributaries at the dividing point between the middle and lower reaches. Bull. Environ. Contam. Toxicol..

[107] Yan H., Zhang C., Zhou Q., Yang S. (2015). Occurrence of perfluorinated alkyl substances in sediment from estuarine and coastal areas of the East China Sea. Environ. Sci. Pollut. Res..

[108] Yao Y., Zhu H., Li B., Hu H., Zhang T., Yamazaki E., Taniyasu S., Yamashita N., Sun H. (2014). Distribution and primary source analysis of per-and poly-fluoroalkyl substances with different chain lengths in surface and groundwater in two cities, North China. Ecotoxicol. Environ. Saf..

[109] Kwok K.Y., Wang X.H., Ya M., Li Y., Zhang X.H., Yamashita N., Lam J.C., Lam P.K. (2015). Occurrence and distribution of conventional and new classes of per-and polyfluoroalkyl substances (PFASs) in the South China Sea. J. Hazard. Mater..

[110] Chen H., Wang X., Zhang C., Sun R., Han J., Han G., Yang W., He X. (2017). Occurrence and inputs of perfluoroalkyl substances (PFASs) from rivers and drain outlets to the Bohai Sea, China. Environ. Pollut..

[111] Dai Z., Zeng F. (2019). Distribution and bioaccumulation of perfluoroalkyl acids in Xiamen coastal waters. J. Chem..

[112] Lee Y.-M., Lee J.-Y., Kim M.-K., Yang H., Lee J.-E., Son Y., Kho Y., Choi K., Zoh K.-D. (2020). Concentration and distribution of per-and polyfluoroalkyl substances (PFAS) in the Asan Lake area of South Korea. J. Hazard. Mater..

[113] Cui W.J., Peng J.X., Tan Z.J., Zhai Y.X., Guo M.M., Li Z.X., Mou H.J. (2019). Pollution Characteristics of Perfluorinated Alkyl Substances (PFASs) in Seawater, Sediments, and Biological Samples from Jiaozhou Bay, China. Huan Jing Ke Xue.

[114] Shi Y., Pan Y., Wang J., Cai Y. (2012). Distribution of perfluorinated compounds in water, sediment, biota and floating plants in Baiyangdian Lake, China. J. Environ. Monit..

[115] Guo M., Zheng G., Peng J., Meng D., Wu H., Tan Z., Li F., Zhai Y. (2019). Distribution of perfluorinated alkyl substances in marine shellfish along the Chinese Bohai Sea coast. J. Environ. Sci. Health.

[116] Li P., Oyang X., Zhao Y., Tu T., Tian X., Li L., Zhao Y., Li J., Xiao Z. (2019). Occurrence of perfluorinated compounds in agricultural environment, vegetables, and fruits in regions influenced by a fluorine-chemical industrial park in China. Chemosphere.

[117] Jin Q., Zhang Y., Gu Y., Shi Y., Cai Y. (2024). Bioaccumulation of legacy and emerging per-and polyfluoroalkyl substances in hydroponic lettuce and risk assessment for human exposure. J. Environ. Sci..

[118] Qian S., Lu H., Xiong T., Zhi Y., Munoz G., Zhang C., Li Z., Liu C., Li W., Wang X. (2023). Bioaccumulation of per-and polyfluoroalkyl substances (PFAS) in ferns: Effect of PFAS molecular structure and plant root characteristics. Environ. Sci. Technol..

[119] Liu Z., Lu Y., Shi Y., Wang P., Jones K., Sweetman A.J., Johnson A.C., Zhang M., Zhou Y., Lu X. (2017). Crop bioaccumulation and human exposure of perfluoroalkyl acids through multi-media transport from a mega fluorochemical industrial park, China. Environ. Int..

[120] Liu Z., Lu Y., Song X., Jones K., Sweetman A.J., Johnson A.C., Zhang M., Lu X., Su C. (2019). Multiple crop bioaccumulation and human exposure of perfluoroalkyl substances around a mega fluorochemical industrial park, China: Implication for planting optimization and food safety. Environ. Int..

[121] Zafeiraki E., Costopoulou D., Vassiliadou I., Leondiadis L., Dassenakis E., Hoogenboom R.L., van Leeuwen S.P. (2016). Perfluoroalkylated substances (PFASs) in home and commercially produced chicken eggs from the Netherlands and Greece. Chemosphere.

[122] Zafeiraki E., Gebbink W.A., Hoogenboom R.L., Kotterman M., Kwadijk C., Dassenakis E., van Leeuwen S.P. (2019). Occurrence of perfluoroalkyl substances (PFASs) in a large number of wild and farmed aquatic animals collected in the Netherlands. Chemosphere.

[123] Kwadijk C.J.A.F., Korytar P., Koelmans A.A. (2010). Distribution of perfluorinated compounds in aquatic systems in the Netherlands. Environ. Sci. Technol..

[124] Hölzer J., Göen T., Just P., Reupert R., Rauchfuss K., Kraft M., Müller J., Wilhelm M. (2011). Perfluorinated compounds in fish and blood of anglers at Lake Mohne, Sauerland area, Germany. Environ. Sci. Technol..

[125] Couderc M., Poirier L., Zalouk-Vergnoux A., Kamari A., Blanchet-Letrouvé I., Marchand P., Vénisseau A., Veyrand B., Mouneyrac C., Le Bizec B. (2015). Occurrence of POPs and other persistent organic contaminants in the European eel (Anguilla anguilla) from the Loire estuary, France. Sci. Total Environ..

[126] Giari L., Guerranti C., Perra G., Lanzoni M., Fano E.A., Castaldelli G. (2015). Occurrence of perfluorooctanesulfonate and perfluorooctanoic acid and histopathology in eels from north Italian waters. Chemosphere.

[127] Pignotti E., Casas G., Llorca M., Tellbüscher A., Almeida D., Dinelli E., Farré M., Barceló D. (2017). Seasonal variations in the occurrence of perfluoroalkyl substances in water, sediment and fish samples from Ebro Delta (Catalonia, Spain). Sci. Total Environ..

[128] Zabaleta I., Bizkarguenaga E., Prieto A., Ortiz-Zarragoitia M., Fernández L.A., Zuloaga O. (2015). Simultaneous determination of perfluorinated compounds and their potential precursors in mussel tissue and fish muscle tissue and liver samples by liquid chromatography–electrospray-tandem mass spectrometry. J. Chromatogr. A.

[129] Munschy C., Olivier N., Veyrand B., Marchand P. (2015). Occurrence of legacy and emerging halogenated organic contaminants in marine shellfish along French coasts. Chemosphere.

[130] Vassiliadou I., Costopoulou D., Kalogeropoulos N., Karavoltsos S., Sakellari A., Zafeiraki E., Dassenakis M., Leondiadis L. (2015). Levels of perfluorinated compounds in raw and cooked Mediterranean finfish and shellfish. Chemosphere.

[131] Bossi R., Strand J., Sortkjær O., Larsen M.M. (2008). Perfluoroalkyl compounds in Danish wastewater treatment plants and aquatic environments. Environ. Int..

[132] Nania V., Pellegrini G.E., Fabrizi L., Sesta G., De Sanctis P., Lucchetti D., Di Pasquale M., Coni E. (2009). Monitoring of perfluorinated compounds in edible fish from the Mediterranean Sea. Food Chem..

[133] Schmidt N., Fauvelle V., Castro-Jiménez J., Lajaunie-Salla K., Pinazo C., Yohia C., Sempere R. (2019). Occurrence of perfluoroalkyl substances in the Bay of Marseille (NW Mediterranean Sea) and the Rhône River. Mar. Pollut. Bull..

[134] Squadrone S., Ciccotelli V., Prearo M., Favaro L., Scanzio T., Foglini C., Abete M.C. (2015). Perfluorooctane sulfonate (PFOS) and perfluorooctanoic acid (PFOA): Emerging contaminants of increasing concern in fish from Lake Varese, Italy. Environ. Monit. Assess..

[135] Houtz E.F., Higgins C.P., Field J.A., Sedlak D.L. (2013). Persistence of perfluoroalkyl acid precursors in AFFF-impacted groundwater and soil. Environ. Sci. Technol..

[136] Bräunig J., Baduel C., Heffernan A., Rotander A., Donaldson E., Mueller J.F. (2017). Fate and redistribution of perfluoroalkyl acids through AFFF-impacted groundwater. Sci. Total Environ..

[137] Allinson M., Yamashita N., Taniyasu S., Yamazaki E., Allinson G. (2019). Occurrence of perfluoroalkyl substances in selected Victorian rivers and estuaries: An historical snapshot. Heliyon.

[138] Schrenk D., Cartus A. (2017). Chemical Contaminants and Residues in Food.

[139] Valsecchi S., Babut M., Mazzoni M., Pascariello S., Ferrario C., De Felice B., Bettinetti R., Veyrand B., Marchand P., Polesello S. (2021). Per-and polyfluoroalkyl substances (PFAS) in fish from European lakes: Current contamination status, sources, and perspectives for monitoring. Environ. Toxicol. Chem..

[140] Arioli F., Ceriani F., Nobile M., Vigano’ R., Besozzi M., Panseri S., Chiesa L.M. (2019). Presence of organic halogenated compounds, organophosphorus insecticides and polycyclic aromatic hydrocarbons in meat of different game animal species from an Italian subalpine area. Food Addit. Contam. Part A.

[141] Barola C., Moretti S., Giusepponi D., Paoletti F., Saluti G., Cruciani G., Brambilla G., Galarini R. (2020). A liquid chromatography-high resolution mass spectrometry method for the determination of thirty-three per-and polyfluoroalkyl substances in animal liver. J. Chromatogr. A.

[142] Fair P.A., Wolf B., White N.D., Arnott S.A., Kannan K., Karthikraj R., Vena J.E. (2019). Perfluoroalkyl substances (PFASs) in edible fish species from Charleston Harbor and tributaries, South Carolina, United States: Exposure and risk assessment. Environ. Res..

[143] Spaan K.M., van Noordenburg C., Plassmann M.M., Schultes L., Shaw S., Berger M.L., Heide-Jørgensen M.P., Rosing-Asvid A., Granquist S.M., Dietz R. (2020). Fluorine mass balance and suspect screening in marine mammals from the northern hemisphere. Environ. Sci. Technol..

[144] Gao Y., Li X., Li X., Zhang Q., Li H. (2018). Simultaneous determination of 21 trace perfluoroalkyl substances in fish by isotope dilution ultrahigh performance liquid chromatography tandem mass spectrometry. J. Chromatogr. B.

[145] Schultes L., Sandblom O., Broeg K., Bignert A., Benskin J.P. (2020). Temporal trends (1981–2013) of per-and polyfluoroalkyl substances and total fluorine in Baltic cod (Gadus morhua). Environ. Toxicol. Chem..

[146] He X., Dai K., Li A., Chen H. (2015). Occurrence and assessment of perfluorinated compounds in fish from the Danjiangkou reservoir and Hanjiang river in China. Food Chem..

[147] Zhou Y., Lian Y., Sun X., Fu L., Duan S., Shang C., Jia X., Wu Y., Wang M. (2019). Determination of 20 perfluoroalkyl substances in greenhouse vegetables with a modified one-step pretreatment approach coupled with ultra performance liquid chromatography tandem mass spectrometry (UPLC-MS-MS). Chemosphere.

[148] Jianchao L., Yinuo X., Jinghua R., Lei H., Chenyang J., Guanghua L., Jun H., Wenliang J. (2025). Occurrence characteristics, source analysis and ecological risk of PFASs in different cultivated soil at an urban scale in Yangtze River Basin. Emerg. Contam..

[149] Dalahmeh S., Tirgani S., Komakech A.J., Niwagaba C.B., Ahrens L. (2018). Per-and polyfluoroalkyl substances (PFASs) in water, soil and plants in wetlands and agricultural areas in Kampala, Uganda. Sci. Total Environ..

[150] Vaccher V., Ingenbleek L., Adegboye A., Hossou S.E., Koné A.Z., Oyedele A.D., Kisito C.S.K., Dembélé Y.K., Hu R., Malak I.A. (2020). Levels of persistent organic pollutants (POPs) in foods from the first regional Sub-Saharan Africa Total Diet Study. Environ. Int..

[151] Kedikoglou K., Costopoulou D., Vassiliadou I., Leondiadis L. (2019). Preliminary assessment of general population exposure to perfluoroalkyl substances through diet in Greece. Environ. Res..

[152] Sonne C., Vorkamp K., Galatius A., Kyhn L., Teilmann J., Bossi R., Søndergaard J., Eulaers I., Desforges J.-P., Siebert U. (2019). Human exposure to PFOS and mercury through meat from baltic harbour seals (Phoca vitulina). Environ. Res..

[153] U.S Environmental Protection Agency (EPA) (2024). Per- and Polyfluoroalkyl Substances (PFAS)|Final PFAS National Primary Drinking Water Regulation. https://www.epa.gov/sdwa/and-polyfluoroalkyl-substances-pfas.

[154] Where Do PFAS Come From?. https://www.inogenalliance.com/blog-post/faq-pfas-definition-sources-benefits-and-risks.

[155] U.S. Food and Drug Administration (FDA) (2024). Per- and Polyfluoroalkyl Substances (PFAS). https://www.fda.gov/food/environmental-contaminants-food/and-polyfluoroalkyl-substances-pfas.

[156] CDC (2024). PFAS and Worker Health. https://www.cdc.gov/niosh/pfas/about/index.html.

[157] Folorunsho O., Kizhakkethil J.P., Bogush A., Kourtchev I. (2024). Effect of short-term sample storage and preparatory conditions on losses of 18 per- and polyfluoroalkyl substances (PFAS) to container materials. Chemosphere.

[158] Lee J.C., Neonaki M., Alexopoulos A., Varzakas T. (2023). Case Studies of Small-Medium Food Enterprises around the World: Major Constraints and Benefits from the Implementation of Food Safety Management Systems. Foods.

[159] Marandi B. (2021). Impacts of Food Contact Chemicals on Human Health. https://www.linkedin.com/posts/ben-marandi_impact-of-migration-of-food-contact-components-activity-7231717007576948737-8SIV?utm_source=share&utm_medium=member_desktop.

[160] Matamoros V., Díez S., Cañameras N., Comas J., Bayona J.M. (2019). Occurrence and human health implications of chemical contaminants in vegetables grown in peri-urban agriculture. Environ. Int..

[161] Minet L., Wang Z., Shalin A., Bruton T.A., Blum A., Peaslee G.F., Schwartz-Narbonne H., Whitehead H., Wu Y., Diamond M.L. (2022). Use and release of per- and polyfluoroalkyl substances (PFASs) in consumer food packaging the in U.S. and Canada. Environ. Sci. Process. Impacts.

[162] Phelps D., Geueke B., Venier M., Scheringer M., Diamond M.L. (2024). Overview of use, migration, and hazards of PFAS in food contact materials. Environ. Sci. Technol..

[163] Onyeaka H., Ghosh S., Obileke K., Miri T., Odeyemi O.A., Nwaiwu O., Tamasiga P. (2024). Preventing chemical contaminants in food: Challenges and prospects for safe and sustainable food production. Food Control..

[164] Ottaway B., Jennings S. (2021). Chemical Contaminants of Food.

[165] Seltenrich N. (2020). PFAS in food packaging: A hot, greasy exposure. Environ. Health Perspect..

[166] Noons N. (2024). Analyzing PFAS Concentrations Along the Blackstone River Watershed. Ph.D. Dissertation.

[167] Food and Drug Administration (FDA) (2024). Environmental Contaminants in Food. https://www.fda.gov/food/chemical-contaminants-pesticides/environmental-contaminants-food.

[168] Environmental Health News (EHN) (2022). IN-DEPTH: What We Know About PFAS in Our Food. https://www.ehn.org/pfas-in-food-2657507160.html.

[169] European Food Safety Authority (EFSA) (2024). Endocrine Active Substances. https://www.efsa.europa.eu/en/topics/topic/endocrine-active-substances.

[170] World Health Organization (WHO) (2022). WHO Global Strategy for Food Safety 2022–2030. https://www.who.int/publications/b/64838.

[171] Food and Drug Administration (FDA) (2024). Food Chemical Safety. https://www.fda.gov/food/food-ingredients-packaging/food-chemical-safety.

[172] NIFA, USDA National Institute of Food and Agriculture (2024). Sustainable Agriculture. https://www.nifa.usda.gov/topics/sustainable-agriculture.

[173] Britannica (2024). Sustainable Agriculture. https://www.britannica.com/technology/sustainable-agriculture.

[174] Forbes (2023). How Technology Is Working to Improve Food Safety and Combat Food Insecurity. https://www.fsis.usda.gov/science-data/research-priorities.

[175] Li X., Shen X., Jiang W., Xi Y., Li S. (2024). Comprehensive review of emerging contaminants: Detection technologies, environmental impact, and management strategies. Ecotoxicol. Environ. Saf..

[176] FAO (2022). New FAO Report Highlights Possible Benefits and Risks. https://www.fao.org/newsroom/detail/fao-report-future-food-foresight/en.

[177] World Health Organization (WHO) (2024). Food Safety. https://www.rand.org/content/dam/rand/pubs/research_reports/RR2500/RR2519/RAND_RR2519.pdf.

[178] Global Harmonization Initiative (GHI) (2025). Welcome to GHI!. https://www.globalharmonization.net/.

[179] Food Safety and Inspection Service (FSIS) (2024). Food Safety Research Priorities & Studies. https://www.usda.gov/sites/default/files/documents/25-2024-FSIS.pdf.

[180] World Health Organization (WHO) (2024). World Food Safety Day. https://www.who.int/campaigns/world-food-safety-day.

[181] Harvard T.H., Chan School of Public Health (2023). Protecting Against ‘Forever Chemicals’. https://www.hsph.harvard.edu/news/hsph-in-the-news/protecting-against-forever-chemicals/.

[182] Food and Agriculture Organization (FAO) (2019). Globalization Increases Risk of Multi-Country Food Safety Issues. https://www.foodsafetynews.com/2019/07/globalization-increases-risk-of-multi-country-food-safety-issues-fao/.

[183] European Food Safety Authority (EFSA) (2024). Chemical Contaminants in Food and Feed. https://www.efsa.europa.eu/en/topics/topic/chemical-contaminants-food-feed.

[184] Food and Drug Administration (FDA) (2024). Bisphenol, A(BPA): Use in Food Contact Application. https://www.fda.gov/food/food-packaging-other-substances-come-contact-food-information-consumers/bisphenol-bpa-use-food-contact-application.

[185] Ramírez Carnero A., Lestido-Cardama A., Vazquez Loureiro P., Barbosa-Pereira L., Rodríguez Bernaldo de Quirós A., Sendón R. (2021). Presence of Perfluoroalkyl and Polyfluoroalkyl Substances (PFAS) in Food Contact Materials (FCM) and Its Migration to Food. Foods.

[186] Brennan N.M., Evans A.T., Fritz M.K., Peak S.A., von Holst H.E. (2021). Trends in the regulation of per- and polyfluoroalkyl substances (PFAS): A scoping review. Int. J. Environ. Res. Public Health.

[187] U.S Environmental Protection Agency (EPA) (2024). PFAS Alternatives PFAS Strategic Roadmap: EPA’s Commitments to Action 2021–2024|US EPA. https://www.epa.gov/pfas/pfas-strategic-roadmap-epas-commitments-action-2021-2024.

[188] Food Packaging Forum (2025). Alternatives to PFAS Are Available for Many Applications Alternatives to PFAS Are Available for Many Applications|Food Packaging Forum. https://foodpackagingforum.org/news/alternatives-to-pfas-are-available-for-many-applications.

[189] Battelle (2024). Phasing Out “Forever Chemicals Phasing Out “Forever Chemicals”: Finding Alternatives for PFAS. https://inside.battelle.org/blog-details/phasing-out-forever-chemicals-finding-alternatives-for-pfas.

[190] Ackerman Grunfeld D., Gilbert D., Hou J., Jones A.M., Lee M.J., Kibbey T.C., O’Carroll D.M. (2024). Underestimated burden of per- and polyfluoroalkyl substances in global surface waters and groundwaters. Nat. Geosci..

[191] Australian Government (2024). PFAS in Australia. https://www.australia.gov.au/pfas.

[192] National Chemicals Working Group of the Heads of EPAs Australia and New Zealand (2020). PFAS National Environmental Management Plan. https://www.pfas.gov.au/news/national-environmental-management-plan-pfas.

[193] Toxics Free Australia. https://www.toxicsfreeaustralia.org.au/tfa-calls-for-urgent-action-on-pfas-contamination-in-australia/.

[194] Shah A.J., Olotu O.O. (2024). The Impact of PFAS in Australia (Review) (Letter of Authorization on File). https://www.linkedin.com/posts/activity-7263501278813536256-DZDW?utm_source=share&utm_medium=member_desktop.

[195] Kirk M., Smurthwaite K., Bräunig J., Trevenar S., D’Este C., Lucas R., Lal A., Korda R., Clements A., Muellerm J. (2018). 3,4 The PFAS Health Study: Systematic Literature Review. https://nceph.anu.edu.au/files/PFAS%20Health%20Study%20Systematic%20Review_1.pdf.

[196] Ayodele A., Obeng-Gyasi E. (2024). Exploring the Potential Link between PFAS Exposure and Endometrial Cancer: A Review of Environmental and Sociodemographic Factors. Cancers.

[197] Warwick N., Johnson R., Jones P.D. (2024). PFOS Concentrations in Platypus Livers. J. Environ. Sci..

[198] Gregory J., PFAS Contamination in New South Wales (2024). Environmental Science & Technology. https://www.sciencedirect.com/science/article/pii/S0048969724005103.

[199] NHMRC (2022). Australian Drinking Water Guidelines. https://www.nhmrc.gov.au/about-us/publications/australian-drinking-water-guidelines.

[200] Food Standards Australia New Zealand (FSANZ) (2022). PFAS in Food. https://www.foodstandards.gov.au.

[201] Thompson J., Eaglesham G., Mueller J. (2011). Concentrations of PFOS, PFOA and other perfluorinated alkyl acids in Australian drinking water. Chemosphere.

[202] Sciancalepore G., Pietroluongo G., Centelleghe C., Milan M., Bonato M., Corazzola G., Mazzariol S. (2021). Evaluation of per- and poly-fluorinated alkyl substances (PFAS) in livers of bottlenose dolphins (Tursiops truncatus) found stranded along the northern Adriatic Sea. Environ. Pollut..

[203] Hayman N.T., Rosen G., Colvin M.A., Conder J., Jennifer A. (2021). Aquatic toxicity evaluations of PFOS and PFOA for five standard marine endpoints. Arblaster Chemosphere.

[204] Taylor C., Kannan K., French S.S. (2021). PFAS Levels in Australian Marine Mammals. Mar. Pollut. Bull..

[205] Wang Q., Ruan Y., Jin L., Tao L.S.R., Lai H., Li G., Yeung L.W.Y., Leung K.M.Y., Lam P.K.S. (2023). Legacy and Emerging Per- and Polyfluoroalkyl Substances in a Subtropical Marine Food Web: Suspect Screening, Isomer Profile, and Identification of Analytical Interference. Environ. Sci. Technol..

[206] Mikkonen H., Kukkonen J.V.K., Leppänen M.T. (2023). Seasonal Trends in PFAS Burden in Livestock. Environ. Sci. Technol..

[207] Coggan T.L., Neale P.A., Müller J.F. (2019). Guidelines for PFAS Disposal in Australia. Environ. Sci. Technol..

[208] Hepburn E., Madden A., Szabo D.T. (2019). PFAS Handling and Disposal Practices. Sci. Total Environ..

[209] Khair Biek S., Khudur L.S., Ball A.S. (2024). Challenges and Remediation, Strategies for Per- and Polyfluoroalkyl, Substances (PFAS) Contamination in Composting. Sustainability.

[210] EPA South Australia PFAS Contamination and Management Guidelines. https://www.epa.sa.gov.au/community/stay-informed/guidance-for-managing-pfas-in-sa.

[211] Sustainability Matters Call for Ban on Persistent Organic Pollutants. https://www.sustainabilitymatters.net.au/content/environment/article/call-for-ban-on-persistent-organic-pollutants-133876.

[212] The Ocean Cleanup. https://theoceancleanup.com/great-pacific-garbage-patch/.

[213] Hobman E.V., Mankad A., Carter D.J. (2022). Public Support for Synthetic Biology Solutions in Australia. J. Environ. Policy.

[214] Hassan S., Akinwumi I., Li L.Y. (2016). Electrokinetic Bioremediation of Polluted Soils. J. Environ. Manag..

[215] Liu X., Zubair M., Kong L., Shi Y., Zhou H., Tong L., Zhu R., Lv Y., Li Z. (2023). Shifts in bacterial diversity characteristics during the primary and secondary fermentation stages of bio-compost inoculated with effective microorganisms agent. Bioresour. Technol..

[216] Marchetto F., Roverso M., Righetti D., Bogialli S., Filippini F., Bergantino E., Sforza E. (2021). Bioremediation of Per- and Poly-Fluoroalkyl Substances (PFAS) by Synechocystis sp. PCC 6803: A Chassis for a Synthetic Biology Approach. Life.

[217] Jafarinejad S. (2025). A Mini-Review of Full-Scale Drinking Water Treatment Plants for Per-and Polyfluoroalkyl Substances (PFAS) Removal: Possible Solutions and Future Directions. Sustainability.

[218] Department of Defence PFAS Investigation Program. https://www.defence.gov.au/environment/pfas.

[219] Maruzzo A.J., Hernandez A.B., Swartz C.H., Liddie J.M., Schaider L.A. (2025). Socioeconomic Disparities in Exposures to PFAS and Other Unregulated Industrial Drinking Water Contaminants in US Public Water Systems. Environ. Health Perspect..

[220] Mueller R., Salvatore D., Brown P., Cordner A. (2024). Quantifying disparities in per-and polyfluoroalkyl substances (PFAS) levels in drinking water from overburdened communities in New Jersey, 2019–2021. Environ. Health Perspect..

[221] Contact (2018). Senate Investigation into PFAS Contamination. https://www.congress.gov/115/chrg/CHRG-115shrg33955/CHRG-115shrg33955.pdf.

[222] Ng C., Cousins I.T., DeWitt J.C., Glüge J., Goldenman G., Herzke D., Lohmann R., Miller M., Patton S., Scheringer M. (2021). Addressing Urgent Questions for PFAS in the 21st Century. Environ. Sci. Tech..

[223] World Bank (2024). Vietnam Overview. https://www.worldbank.org/en/country/vietnam/overview.

[224] United Nations Development Programme (2016). Report 10 Years of Implementing Stockholm Convention on Persistent Organic Pollutants in Viet Nam 2005–2015. https://www.undp.org/vietnam/publications/report-10-years-implementing-stockholm-convention-persistent-organic-pollutants-viet-nam-2005-2015.

[225] Enviliance Asia (2022). Vietnam enacts Decree on POPs Control under the Environmental Protection Law 2020. https://enviliance.com/regions/southeast-asia/vn/report_5390.

[226] PanNature (2019). Vietnam’s PFAS Situation Report. https://www.nature.org.vn/en/2019/05/vietnams-pfas-situation-report/.

[227] Vietnam National Assembly (2017). National Plan for the Implementation of the Stockholm Convention on Persistent Organic Pollutants by 2025 with a Vision to 2030. https://en.qdnd.vn/politics/news/national-plan-for-stockholm-convention-implementation-issued-485908.

[228] EPA U., Persistent Organic Pollutants: A Global Issue (2014). A Global Response. https://www.who.int/news-room/questions-and-answers/item/food-safety-persistent-organic-pollutants-(pops).

[229] IPEN (2020). Global Action to Eliminate Toxic Pollutants. https://ipen.org.

[230] Sun Q., Bi R., Wang T., Su C., Chen Z., Diao J., Zheng Z., Liu W. (2021). Are there risks induced by novel and legacy poly- and perfluoroalkyl substances in coastal aquaculture base in South China?. Sci. Total Environ..

[231] USEPA (2016). Health Effects Support Document for Perfluorooctanoic Acid (PFOA). U.S. https://www.epa.gov/sites/default/files/2016-05/documents/pfoa_hesd_final-plain.pdf.

[232] Zhao S., Xia X., Yang J. (2011). Bioaccumulation and biomagnification of poly- and perfluoroalkyl substances in marine food webs from Bohai Sea, China. Environ. Pollut..

[233] Xie S., Zhao J., Zhang J., Hou X., Cai Z. (2019). PFASs in aquatic species from an urbanized river in the Pearl River Delta, South China. Environ. Pollut..

[234] Fiedler H., Kallenborn R., De Boer J., Sydnes L.K. (2019). The Stockholm convention: A tool for the global regulation of persistent organic pollutants. Chem. Int..

[235] ASEAN (2018). ASEAN POPs Protocol. https://asean.org/pops-protocol.

[236] World Bank (2020). Funding for PFAS-Related Projects. https://www.worldbank.org.

[237] Government of Canada (2023). Chemicals Management Plan. https://www.canada.ca/en/health-canada/services/chemical-substances/chemicals-management-plan.html.

[238] Environment and Climate Change Canada ECCC (2023). Risk Management Scope for Per- and Polyfluoroalkyl Substances (PFAS). https://www.canada.ca/en/services/environment/climatechange.html.

[239] University of Toronto (2024). Four Ontario Universities Funded to Research Impacts of PFAS, 6PPD in Great Lakes. http://esemag.com.

[240] Health Canada (2024). Objective for Canadian Drinking Water Quality Per- and Polyfluoroalkyl Substances. https://www.canada.ca/en/health-canada/services/publications/healthy-living/objective-drinking-water-quality-per-polyfluoroalkyl-substances.html.

[241] Canadian Institute of Food Safety CIFS Innovations in Food Safety Technology to Watch for in 2022. https://blog.foodsafety.ca/innovations-food-safety-technology-2022.

[242] Government of Canada (2024). Persistent Organic Pollutants: Stockholm Convention. https://www.canada.ca/en/environment-climate-change/services/canadian-environmental-protection-act-registry/publications/update-canada-national-implementation-plan-stockholm-convention-persistent-organic-pollutants.html.

[243] Health Canada (2024). Pest Control Products Act. https://www.canada.ca/en/health-canada/services/consumer-product-safety/pesticides-pest-management/public/protecting-your-health-environment/pest-control-products-acts-and-regulations-en.html.

[244] Government of Canada (2024). Prohibition of Certain Toxic Substances Regulations, 2012. https://www.canada.ca/en/environment-climate-change/services/management-toxic-substances/prohibition-regulations.html.

[245] Environment and Climate Change Canada (ECCC) (2024). Wastewater Systems Effluent Regulations. https://www.canada.ca/en/environment-climate-change/services/wastewater/system-effluent-regulations-reporting.html.

[246] Government of Canada (2024). Updated Draft State of Per- and Polyfluoroalkyl Substances (PFAS) Report. https://www.canada.ca/en/environment-climate-change/services/evaluating-existing-substances/updated-draft-state-per-polyfluoroalkyl-substances-report.html.

[247] OECD (2024). Global Forum on the Environment Dedicated to Per- and Polyfluoroalkyl Substances (PFAS). https://www.oecd.org/en/events/2024/02/global-forum-environment-per-and-polyfluoroalkyl-substances.html.

[248] Government of Canada (2023). Draft State of per- and Polyfluoroalkyl Substances (PFAS) Report. Report.

[249] Health Canada (2023). Risk Management Scope for Per- and Polyfluoroalkyl Substances (PFAS). https://www.canada.ca/en/environment-climate-change/services/evaluating-existing-substances/risk-management-scope-per-polyfluoroalkyl-substances.html.

[250] CIHR Canadian Institutes of Health Research (2024). Research on PFAS. https://cihr-irsc.gc.ca.

[251] NCP Northern Contaminants Program (2024). Northern Contaminants Program 2024 Call for Proposals. https://science.gc.ca.

[252] Government of Canada (2023). Government of Canada Taking Next Step in Addressing “Forever Chemicals” PFAS. https://www.canada.ca/en/environment-climate-change/news/2023/05/government-of-canada-taking-next-step-in-addressing-forever-chemicals-pfas.html.

[253] (2019). List of Bases Contaminated with PFAS Chemicals Expected to Grow, Pentagon Says. https://military.com.

[254] Forever Pollution Project (2023). The Forever Pollution Project. https://foreverpollution.eu/.

[255] University of Birmingham (2024). Researchers Discover 10 PFAS Chemicals in Drinking Water Across UK and China. https://www.birmingham.ac.uk/news/2024/forever-chemicals-found-in-bottled-and-tap-water-from-around-the-world.

[256] European Chemical Agency ECHA (2023). Per- and Polyfluoroalkyl Substances (PFAS). https://echa.europa.eu/hot-topics/perfluoroalkyl-chemicals-pfas.

[257] European Commission (EC) (2024). Research and Innovation—Per- and Polyfluorinated Substances (PFAS). https://ec.europa.eu/newsroom/rtd/items/821889/.

[258] Das R., Ananthanarasimhan J., Rao L. “PFAS” Exploring the Origins, Impact, Regulations and Remediation Technologies—An Overview. https://link.springer.com/article/10.1007/s41745-024-00442-8.

[259] European Environment Agency (EEA) (2023). Emerging Chemical Risks in Europe PFAS. https://www.eea.europa.eu/en/analysis/publications/emerging-chemical-risks-in-europe.

[260] ChemSec (2023). The Top 12 PFAS Producers in the World and the Staggering Societal Costs of PFAS Pollution. https://chemsec.org/reports/the-top-12-pfas-producers-in-the-world-and-the-staggering-societal-costs-of-pfas-pollution/.

[261] European Parliament (2020). Growing Number of Illegal Landfills Across the EU. https://www.europarl.europa.eu/portal/en.

[262] Plastics Waste Challenge (PWC) (2019). The Road to Circularity. https://www.pwc.at/de/publikationen/klimawandel-nachhaltigkeit/pwc-circular-economy-study-2019.pdf.

[263] PlasticsEurope (2019). Plastics—The Facts 2019. https://plasticseurope.org/knowledge-hub/plastics-the-facts-2019/.

[264] The World Economic Forum (2016). The New Plastics Economy: Rethinking the Future of Plastics. https://www.weforum.org/publications/the-new-plastics-economy-rethinking-the-future-of-plastics/.

[265] European Commission (EC) (2024). A European Strategy for Plastics in a Circular Economy. https://eur-lex.europa.eu/legal-content/EN/TXT/?uri=COM:2018:28:FIN.

[266] Geyer R., Jambeck J.R., Law K.L. (2017). Production, use, and fate of all plastics ever made. Sci. Adv..

[267] EU Science Hub (2024). Less Than One-Fifth of EU Plastic Was Recycled in 2019, but 2025 Targets Can Be Still Reached. https://joint-research-centre.ec.europa.eu/jrc-news-and-updates/less-one-fifth-eu-plastic-was-recycled-2019-2025-targets-can-be-still-reached-2024-01-25_en.

[268] EC Europa (2024). Food Contact Materials. https://food.ec.europa.eu/food-safety/chemical-safety/food-contact-materials_en.

[269] Bluefield Research (2022). US$6.15 Billion PFAS Remediation Forecast Underpinned by Changing Regulatory Environment. https://www.bluefieldresearch.com/ns/us6-15-billion-pfas-remediation-forecast-underpinned-by-changing-regulatory-environment/.

[270] Source Intelligence U.S (2024). PFAS Regulations by State. https://blog.sourceintelligence.com/pfas-regulations-how-to-remain-compliant.

[271] NIEHS (National Institute of Environmental Health Sciences) (2022). Plant-Based Material Can Remediate PFAS, New Research Suggests. https://factor.niehs.nih.gov/2022/9/science-highlights/pfas-remediation.

[272] (2021). H.R.2467—PFAS Action Act of 2021. https://www.congress.gov/bill/117th-congress/house-bill/2467.

[273] Lee J.C., Agriopoulou S., Varzakas T. (2024). Pathways to Implementing Food Systems-Capacity Building Programs for Smallholder Farmers: Major Constraints and Benefits. https://digitaledition.food-safety.com/june-july-2024/column-management/.

[274] U.S. Department of Agriculture (USDA) (2024). USDA Developing a Roadmap to Tackle PFAS on Farmland. https://www.agriculturedive.com/news/usda-roadmap-pfas-farmland-forever-chemicals/730770/.

[275] The Natural Resources Defense Council (NRDC) (2024). Toxic Drinking Water: Addressing the PFAS Contamination Crisis. https://www.nrdc.org/sites/default/files/2024-10/PFAS_Toxic_Drinking_Water_FS_24-09-B_07.pdf.

[276] Huang X., Wei X., Liu H., Li W., Shi D., Qian S., Sun W., Yue D., Wang X. (2022). Occurrence of per-and polyfluoroalkyl substances (PFAS) in municipal solid waste landfill leachates from western China. Environmental Science and Pollution Research.

[277] American Chemical Society (ACS) (2024). Some Landfill ’Burps’ Contain Airborne PFAS, Study Finds. https://www.acs.org/pressroom/presspacs/2024/june/some-landfill-burps-contain-airborne-pfas-study-finds.html.

[278] Hagarty A. (2023). United States Analysis of the Regulatory Inception of Per-and Polyfluoroalkyl Substances (PFAS) in Drinking Water Policy Among States and Review of Regulatory Efforts Made by the Federal Environmental Protection Agency.

[279] Ehsan M.N., Riza M., Pervez M.N., Li C.-W., Zorpas A.A., Naddeo V. (2024). PFAS contamination in soil and sediment: Contribution of sources and environmental impacts on soil biota. Case Stud. Chem. Environ. Eng..

[280] Jensen C.R., Genereux D.P., Solomon D.K., Knappe D.R.U., Gilmore T.E. (2024). Forecasting and Hindcasting PFAS Concentrations in Groundwater Discharging to Streams near a PFAS Production Facility. Environ. Sci. Technol..

[281] College of Sciences Research and Innovation COSRI (2024). It Could Take Over 40 Years for PFAS to Leave Groundwater. https://sciences.ncsu.edu/news/it-could-take-over-40-years-for-pfas-to-leave-groundwater/.

[282] (2024). Study Finds It Could Take Over 40 Years to Flush PFAS Out of Groundwater. https://phys.org/news/2024-10-years-flush-pfas-groundwater.pdf.

[283] Lee J.C., Daraba A., Voidarou C., Rozos G., Enshasy H.A.E., Varzakas T. (2021). Implementation of Food Safety Management Systems along with Other Management Tools (HAZOP, FMEA, Ishikawa, Pareto). The Case Study of Listeria monocytogenes and Correlation with Microbiological Criteria. Foods.

[284] Governing (2024). Legislative Efforts Against Forever Chemicals Grow Across Nation. https://www.governing.com/policy/legislative-efforts-against-forever-chemicals-grow-across-nation.

[285] Manojkumar Y., Pilli S., Rao P.V., Tyagi R.D. (2023). Sources, occurrence and toxic effects of emerging per- and polyfluoroalkyl substances (PFAS). Neurotoxicol. Teratol..

[286] MERCOSUR (Mercado Común del Sur). https://www.mercosur.int/en/.

[287] Inter-American Development Bank (IDB). https://www.iadb.org/en.

[288] United Nations Environment Programme (UNEP) Per- and Polyfluoroalkyl Substances (PFASs)|UNEP—UN Environment Programme Five African Countries Unite to Reduce Release of Hazardous Chemicals from Plastics. https://www.unep.org/.

[289] Stockholm Convention on Persistent Organic Pollutants (POPs) What Are POPs. https://www.pops.int/TheConvention/ThePOPs/tabid/673/Default.aspx.

[290] Li X., Wang Y., Cui J., Shi Yali Y.C. (2024). Occurrence and fate of per- and polyfluoroalkyl substances (PFAS) in atmosphere: Size-dependent gas-particle partitioning, precipitation scavenging, and amplification. Environ. Sci. Technol..

[291] National Institute of Environmental Health Sciences Perfluoroalkyl and Polyfluoroalkyl Substances (PFAS). https://www.niehs.nih.gov/health/topics/agents/pfc.

[292] Our Current Understanding of the Human Health and Environmental Risks of PFAS. https://www.epa.gov/pfas/our-current-understanding-human-health-and-environmental-risks-pfas.

[293] What to Know About ‘Forever Chemicals,’ Artificial Turf, Phillies Cancer Deaths, and Our Story. https://www.inquirer.com/news/pfas-forever-chemicals-drinking-water-vet-astroturfphiladelphia-20230307.html.

[294] Earth Island Journal Life Along the Banks of One of Latin America’s Most Polluted Waterways. https://www.earthisland.org/journal/index.php/articles/entry/banks-latin-america-most-polluted-waterways.

[295] Associated Press It looks Like a Stream of Blood. A River Near Buenos Aires Turns Red, Sparking Fears of Toxic Leak. https://apnews.com/article/argentina-buenos-“aires-river-red-industrial-leak-41a713c0ecdadadf204c330465a3f7e9.

[296] Global Environment Facility (GEF). https://www.thegef.org/.

[297] Global Recycling. https://global-recycling.info/archives/8156.

[298] Ssebugere P., Sillanpää M., Matovu H., Wang Z., Schramm K.-W., Omwoma S., Wanasolo W., Ngeno E.C., Odongo S. (2020). Environmental levels and human body burdens of per- and poly-fluoroalkyl substances in Africa: A critical review. Sci. Total Environ..

[299] Stockholm Convention on Persistent Organic Pollutants (POPs). https://chm.pops.int/Implementation/IndustrialPOPs/PFAS/Overview/tabid/5221/Default.aspx.

[300] Chokwe T.B., Themba N., Mahlambi P.N., Mngadi S.V., Sibali L.L. (2024). Poly- and per-fluoroalkyl substances (PFAS) in the African environments: Progress, challenges, and future perspectives. Environ. Sci. Pollut. Res..

[301] Akinrinade O.E., Agunbiade F.O., Alani R.A., Ayejuyo O. (2024). Implementation of the Stockholm Convention on persistent organic pollutants (POPs) in Africa—Progress, challenges, and recommendations after 20 years. Environ. Sci. Adv..

[302] Department of Forestry, Fisheries and the Environment (DFFE) National Implementation Plan for the Stockholm Convention on Persistent Organic Pollutants. South Africa. https://www.thedtic.gov.za/wp-content/uploads/NIP2012.pdf.

[303] African Union (AU). https://au.int/.

[304] European Environment Agency What Are PFAS and How Are They Dangerous for My Health?. https://www.eea.europa.eu/en/about/contact-us/faqs/what-are-pfas-and-how-are-they-dangerous-for-my-health.

[305] Li J., Li X., Da Y., Yu J., Long B., Zhang P., Bakker C., McCarl B.A., Yuan J.S., Dai S.Y. (2022). Sustainable environmental remedi-ation via biomimetic multifunctional lignocellulosic nano-framework. Nat. Commun..

[306] Kelbessa W., Constance C. (2022). Environmental Injustice and Disposal of Hazardous Waste in Africa. The Palgrave Handbook of Global Sustainability.

[307] Heinrich H.W. (1931). Industrial Accident Prevention: A Scientific Approach. https://archive.org/details/dli.ernet.14601.

[308] Shabani T., Jerie S., Shabani T. (2023). A Comprehensive Review of the Swiss Cheese Model in Risk Management. Saf. Extrem. Environ..

[309] Tague N.R. (2005). The Quality Toolbox, Third Edition—ASQ. https://asq.org/quality-press/display-item?item=H1592.

[310] Leveson N. (2011). Engineering a Safer World: Systems Thinking Applied to Safety. https://direct.mit.edu/books/oa-monograph/2908/Engineering-a-Safer-WorldSystems-Thinking-Applied.

[311] Reason J. (1990). Human Error. https://www.cambridge.org/highereducation/books/human-error/281486994DE4704203A514F7B7D826C0.

[312] Ishikawa K., Asian Productivity Organization (1968). Guide to Quality Control. https://books.google.gr/books/about/Guide_to_Quality_Control.html?id=POEeAQAAIAAJ&redir_esc=y:.

[313] Andersen B., Fagerhaug T. (2006). Root Cause Analysis: Simplified Tools and Techniques.

[314] Grandjean P. (2007). The impact of environmental chemicals on human health: A review of the evidence. Environ. Health Perspect..

[315] Post G. (2010). Per- and polyfluoroalkyl substances (PFAS): A review of their environmental and health impacts. Environ. Sci. Technol..

[316] Ishikawa K. (1976). Guide to Quality Control.

[317] U.S. Environmental Protection Agency (2021). Per- and Polyfluoroalkyl Substances (PFAS) in Wastewater. https://www.epa.gov/pfas.

[318] Tague N.R. (2005). The Quality Toolbox. https://www.amazon.com/Quality-Toolbox-2nd-Nancy-Tague/dp/0873898710.

[319] Buck R.C., Franklin J., Berger U., Conder J.M., Cousins I.T., de Voogt P., Jensen A.A., Kannan K., Mabury S.A., van Leeuwen S.P. (2011). Perfluoroalkyl and polyfluoroalkyl substances in the environment: Terminology, classification, and origins. Integr. Environ. Assess. Manag..

[320] Lindstrom A.B., Strynar M.J., Libelo E.L. (2011). Polyfluorinated compounds: Past, present, and future. Environ. Sci. Technol..

[321] Lau C., Anitole K., Hodes C., Lai D., Pfahles-Hutchens A., Seed J. (2007). Perfluoroalkyl acids: A review of monitoring and toxicological findings. Toxicol. Sci..

[322] Newton S., McMahen R., Stoeckel J., Chislock M., Lindstrom A., Strynar M. (2020). Novel polyfluorinated compounds identified using high resolution mass spectrometry downstream of manufacturing facilities near Decatur, Alabama. Environ. Sci. Technol..

[323] Orellana R., Cumsille A., Piña-Gangas P., Rojas C., Arancibia A., Donghi S., Stuardo C., Cabrera P., Arancibia G., Cárdenas F. (2022). Economic Evaluation of Bioremediation of Hydrocarbon-Contaminated Urban Soils in Chile. Sustainability.

[324] (2024). Bioremediation in Sustainable Wastewater Management. https://link.springer.com/book/10.1007/978-981-99-2560-5.

[325] Waterkeeper Alliance (2024). Empowering Communities and Spurring Governmental Action to Stop and Clean Up PFAS Pollution. https://sdgs.un.org/partnerships/empowering-communities-and-spurring-governmental-action-stop-and-clean-pfas-pollution.

[326] Grandjean P., Clapp R. (2015). Perfluorinated Alkyl Substances: Emerging Insights into Health Risks. New Solut..

[327] C8 Science Panel (2012). Probable Link Evaluation of PFOA and Human Health. http://www.c8sciencepanel.org/publications.html.

[328] Li J., Sun J., Li P. (2022). Exposure routes, bioaccumulation and toxic effects of per- and polyfluoroalkyl substances (PFASs) on plants: A critical review. Environ. Int..

[329] Glüge J., Scheringer M., Cousins I.T., DeWitt J.C., Goldenman G., Herzke D., Lohmann R., Ng C.A., Trier X., Wang Z. (2020). An overview of the uses of per- and polyfluoroalkyl substances (PFAS). Environ. Sci. Process. Impacts.

[330] Kang H., Choi K., Lee H.S., Kim D.H., Park N.Y., Kim S., Kho Y. (2016). Elevated levels of short carbon-chain PFCAs in breast milk among Korean women: Current status and potential challenges. Environ. Res..

[331] Zhou Y., Lin X., Xing Y., Zhang X., Lee H.K., Huang Z. (2023). Per- and polyfluoroalkyl substances in personal hygiene products: The implications for human exposure and emission to the environment. Environ. Sci. Technol..

[332] Susmann H.P., Schaider L.A., Rodgers K.M., Rudel R.A. (2019). Dietary habits related to food packaging and population exposure to PFASs. Environ. Health Perspect..

[333] Kang H., Kim D.H., Choi Y.H. (2024). Elevated levels of serum per- and poly-fluoroalkyl substances (PFAS) in contact lens users of U.S. young adults. Chemosphere.

[334] Segedie L. (2023). Indications of PFAS “Forever Chemicals” in Contact Lenses—Report. Mamavation.

[335] Spyrakis F., Dragani T.A. (2023). The EU’s Per- and Polyfluoroalkyl Substances (PFAS) Ban: A Case of Policy over Science. Toxics.

[336] Adewuyi A., Li Q. (2024). Emergency of per- and polyfluoroalkyl substances in drinking water: Status, regulation, and mitigation strategies in developing countries. Eco-Environ. Health.

[337] Dauchy X., Boiteux V., Colin A., Bach C., Rosin C., Munoz J.-F. (2018). Poly- and Perfluoroalkyl Substances in Runoff Water and Wastewater Sampled at a Firefighter Training Area. Arch. Environ. Contam. Toxicol..

