# Supplementary material for: Research Progress in Current and Emerging Issues of PFASs’ Global Impact: Long-Term Health Effects and Governance of Food Systems
Source: Foods. 2025 Mar 11;14(6):958. doi: 10.3390/foods14060958 (PMC11941069; doi:10.3390/foods14060958)
Supplement: Supplementary file 1 [file foods-14-00958-s001.zip › foods-3465211-supplementary.pdf]

Table S1. PFAS Concentrations in Food and Dietary intake in several countries.

Country	Food products samples	Origin	Assessed PFAS	Mass Spectroscopy Method of PFAS determination	Findings	Total PFAs	References
Freshwater products							
China	1. 16 fish samples<br>2. 25 meat samples<br>3. 3 egg samples<br>4. 72 vegetable samples	Supermarket	22 PFAS	Ultraperformance liquid chromatography–triple quadrupole mass Spectrometry (UPLC-TQS)	-Linear-PFASs and PFBS were frequently identified in fish and vegetables, respectively	1. 168 pg/kg bw<br>2. 54 pg/kg bw<br>3. 47 pg/kg bw<br>4. 46 pg/kg bw	[99]
European subalpine area	Fish<br>1. fillets,<br>2. liver,<br>3. viscera,<br>4. carcasses	Wild	11 PFAS	LC-MS-MS	In a lake, PFAS in fish is linked with the level of urbanization of the lake catchment	1.0.85-13.8 ng/g<br>2. 13.5-36.9 ng/g<br>3. 11.5-59.0 ng/g<br>4. 3.8-31 ng/g	[139]
Italy	Red deer, roe deer, chamois, and wild boar	Wild	16 PFAS	Liquid chromatography coupled to high-resolution mass spectrometry (HRMS) Orbitrap (LCHRMS)	PFAS only in wild boar species	0.83-2.90 ng/g	[140]
Italy	Wild boar	Farm animals	33 PFAS	LC coupled to a hybrid high-resolution mass analyser (LC-Q-Orbitrap)	18 PFAS s were assessed at level > 0.2 ng/g]	-	[141]
China	1. Vegetable<br>2. Egg<br>3. Egg white<br>4. Egg yolk	Fluorochemical industrial park	10 PFAS	HPLC-MS/MS	PFBA and PFBS were the major contaminants in both home-produced vegetables and eggs	1. 1.7-87 ng/g<br>2. 63-108 ng/g<br>3. 43-58 ng/g<br>4. 106-146 ng/g	[100]
USA	1.Fish fillet<br>2. Mullet<br>3. Spot<br>4. Croaker<br>5. Red Drum	Estuary	11 PFAS	HPLC-MS/MS	-PFAS levels in fillets changed by location<br>-In certain species and locations PFOS levels surpassed human screening standards for cancer risk	1. 12.4-12.7 ng/g<br>2. 28.4-33 ng/g<br>3. 19.5-28.4 ng/g<br>4. 27-29.6 ng/g	[142]

						5. 23.4-31.1 ng/g	
Netherlands	22 bivalves, crustaceans, marine and farmed fish.	wild and farmed aquatic animals	16 PFAS	HPLC-MS/MS	PFAS levels in eel> bivalves and crustaceans > marine fish > farmed fish	0.06 -172 ng/g	[122]
Sweden	1. Liver of Killer whales<br>2. Harbor seals<br>3. Ringed seals	Seawater	36 PFAS	HPLC-MS/MS	-PFOS dominated in all but one Icelandic and 3 US samples, where the 7:3 FTCA) was predominant.	1.614 ng/g<br>2. 640 ng/g<br>3. 536 ng/g	[143]
China	Soft tissues of Shellfish	Seawater	35 PFAS	HPLC-MS/MS	- BAFs were linked with C chain length,<br>- BSAF diminished with growth in C chain length (C8-C13).	15.5–27.5 ng/g	[113]
China	1. Chameleon goby Muscle<br>2. Grass carp muscle<br>3. Ghost crab soft tissues<br>4. Hermit crab soft tissues<br>5. Oyster soft tissues	Seawater and Freshwater	6 PFAS	HPLC-MS/MS	PFAS in water is highly positively connected with the PFAS concentration in sediments	1. 10.97–12.93 ng/g<br>2. 8.87–10.66 ng/g<br>3. 7.8–10.47 ng/g<br>4. 7.73–8.06 ng/g<br>5. 12.45–12.76 ng/g	[111]
China	Fish (Sea bream, Pagrosomus major) samples	Seawater	11 PFAS	Ultra High performance liquid chromatography-tandem mass spectrometry (UHPLC-MS/MS)	PFOS was the main compound that was detected at 0.13 ng/g	0.04–2.14 ng/g	[144]
Sweden	Liver of Baltic cod	Seawater	28 PFAS	UHPLC-MS/MS	Significant negative correlations were assessed between PFASs and liver somatic index.<br>-body length was negatively correlated with PFOA and PFNA, and positively linked to perfluoro-n-dodecanoic acid (PFDoDA) and PFOSA	6.03–23.9 ng/g	[145]
Greece	1. Giant devil ray (Muscle and gills)	Seawater	15 PFAS	HPLC-MS/MS	Perfluorotridecanoic acid (PFTrDA) was the most predominant compound in terms of concentration and frequency of detection,	1. 1.5–4.4 ng/g<br>2. 1.5–4.4 ng/g	[121]

	2. Small-tooth sand tiger (Muscle and gills)<br>3. Small-tooth sand tiger (gills and liver)				followed by Perfluoropentanoic acid (PFUnDA) and PFOS	3. 62.2–65.4 ng/g	
USA	1.Whole fish Atlantic croaker<br>2. Red drum<br>3 Spot<br>4. Spotted seatrout<br>5. Striped mullet	Seawater	15 PFAS	HPLC-MS/MS	Low PFAS levels in mullet and highest in croaker, spot, red drum, seatrout, and flounder.<br>PFOS levels surpassed wildlife protective guidelines in 83% of whole fish.	1. 15.2–21.3 ng/g<br>2. 11.3-66.1 ng/g<br>3. 14.7–67.8 ng/g<br>4. 17.3–85.4 ng/g<br>5. 6.2-20.7 ng/g	[142]
China	1. Liver, Muscle fish<br>2. Yellow croaker, Mandarin fish<br>3. Crucian Carp	Freshwater	8 PFAS	UPLC–MS/MS	The calculated hazard ratio (HR) values designate no risk to human health if the amount of fish consumption is less than 14.35 kg ww (2.87 kg dw/ person/ year).	1. 8.99–87.9 ng/g<br>2. 3.02–51.2 ng/g<br><br>3. 3.15–4.09 ng/g	[146]
Agricultural products							
China	1. Shoot vegetables<br>2. Fruit/ vegetables<br>3. Flower vegetables<br>4. Root vegetables<br>5. Grain crops	Agricultural field	12 PFAS	HPLC-MS/MS	Vegetables and grains possess bioaccumulation preference to shorter-chain PFASs.<br>-Vegetables showed the highest BAFs of ∑PFASs in multiple crops.	1. ≈2355 ng/g<br>2. ≈1115 ng/g<br>3. ≈410 ng/g<br>4. ≈333 ng/g<br>5. ≈580 ng/g	[119]
China	Fruit<br>Shoot/stem vegetables (leek, cucumber, eggplant, lettuce, and tomato)<br>Mushroom	Greenhouse	20 PFAS	UPLC-MS-MS	PFOA was detected most frequently at 0.023 - 0.153 mg/ kg<br>-Total PFAS levels extended - 0 to 0.683 mg/kg	(0 – 0.68ng/g)	[147]
China	Fruit	Greenhouse	8 PFAS	HPLC–MS/MS	Bioaccumulation efficiencies for PFAS from soil to vegetables were negatively related to the C chain length in PFASs.	≈ 93.5 ng/g	[102]

China	Fruit and<br>Shoot vegetables (tomato, cucumber, eggplant, pepper, and Chinese cabbage)	Home gardens	10 PFAS	HPLC–MS/MS	PFAS might be recognized for the irrigation with groundwater from local public water systems	1.7-78 ng/g	[100]
China	21 species	Agricultural field	21 PFAS	HPLC–MS/MS	Short-chain (C ≤ 8) PFCA was presented in 97.1% of all samples	0.3-11.5 ng/g	[148]
Uganda	1.Yam (root)<br>2.Maize (grain)<br>3.Sugarcane (Shoot/stem)	Wetland Lake	26 PFAS	HPLC-MS/MS	PFAS concentrations < those reported in neighboring countries (e.g. Kenya) and industrialized countries (e.g. Germany and USA).	1. 0.36 ng/g<br>2. 0.2 ng/g<br>3. 0.35 ng/g	[149]
Meat and livestock							
Benin, Cameroon, Mali and Nigeria	Cooked beef	NA	14 PFAS	HPLC-MS/MS	PFASs concentrations equivalent or < in previous international Total Diet Study (TDS)	0.4 – 12.05 ng/g	[150]
China	1. Pork<br>2. Pork liver<br>3. Chicken<br>4. Duck<br>5. Beef<br>6. Egg	Supermarket	19 PFAS	HPLC-MS/MS	Linear-PFAS s and PFBS were mainly found in fish and vegetables, respectively	1. 0.02 ng/g<br>2. 0.4 ng/g<br>3. 0.026 ng/g<br>4. 0.25 ng/g<br>5. 0.04 ng/g<br>6. 0.03 ng/g	[99]
Italy	Wild boar	Wild	16 PFAS	UPLC-MS-MS	-FOS was noticed in 25% of the wild boar samples with very low levels.	0.83 – 2.90 ng/g	[140]
China	1. Egg<br>2. Egg white<br>3. Egg yolk	Home-produced	10 PFAS	HPLC–MS/MS	-PFBA was the dominant PFAS contaminant in eggs<br>- PFBS and PFOA contributed 31% and 24% of the whole PFASs, respectively	1. 58 ng/g<br>2. 146 ng/g<br>3. 108 ng/g	[100]
Greece	1. Beef<br>2. Chicken<br>3. Sheep<br>4. Milk<br>5. Eggs	Production sites	2 PFAS	HPLC-MS/MS	Weekly intake values < the acceptable weekly intake proposed by EFSA	6. 1. 0.90 ng/g<br>7. 2. 1 ng/g	[151]

						8. 3. 1.75 ng/g<br>9. 4. 0.1 ng/g<br>10. 5. 0.81 ng/g	
Denmark	Meat of Harbor seals	Wild	15 PFAS	HPLC–MS/MS	PFAS was non-significantly higher in sub-adults than in adults	17.2 – 21.8 ng/g	[152]
